# A novel coupled fluid-behavior model for simulating dynamic huddle formation

**DOI:** 10.1371/journal.pone.0203231

**Published:** 2018-08-31

**Authors:** Wen Gu, Jason K. Christian, C. Brock Woodson

**Affiliations:** College of Engineering, University of Georgia, Athens, GA, United States of America; The Ohio State University, UNITED STATES

## Abstract

A coupled numerical model is developed to examine aggregative behavior in instances where the behavior not only responds to the environment, but the environment responds to the behavior such as fish schooling and penguin huddling. In the coupled model, the full Navier-Stokes equations are solved for the wind field using a finite difference method (FDM), and coupled to a smoothed particle hydrodynamics (SPH) model adapted to simulate animal behavior (penguins are individual particles in the SPH). We use the model to examine the dynamics of penguin huddling as a purely individual fitness maximizing behavior. SPH is a mesh-free Lagrangian method driven by local interactions between neighboring fluid particles and their environment allowing particles to act as free ranging ‘animals’ unconstrained by a computational grid that implicitly interact with one another (a critical element of aggregative behavior). The coupled model is recomputed simultaneously as the huddle evolves over time to update individual particle positions, redefine the properties of the developing huddle (i.e., shape and density), and adjust the wind field flowing through and around the dynamic huddle. This study shows the ability of a coupled model to predict the dynamic properties of penguin huddling, to quantify biometrics of individual particle “penguins”, and to confirm communal penguin huddling behavior as an individualistic behavior.

## 1. Introduction

Formation of animal aggregations has a long history in the study of behavioral ecology with a central debate concerning whether aggregations form due to cooperative behaviors that benefit the entire group, or from individual behaviors that seek only to maximize individual fitness [1]. From an evolutionary standpoint, the latter is more parsimonious because the selection for maximizing fitness is straightforward. However, if all individuals act to maximize their own benefits, there may be no average benefit for the group, and similarly, if all individuals act to maximize group fitness, there is no evolutionary mechanism for the behavior to exist [2,3]. Numerical models have provided unique insights into aggregative behavior [4], but are generally unidirectional (environmental parameters are only passed to behavior model, one-way coupling) [5]. In many cases, such as fish schooling and penguin huddling, the community or individual benefit may arise due to the environment in turn responding to the huddle formation (huddle parameters need to be passed back to environmental model, two-way coupling). To address this issue, we develop a two-way coupled fluid-behavior model to examine aggregative behavior in animals and use huddling behavior in emperor penguins as a case study. We then use the model to assess the hypothesis of whether behaviors that maximize individual fitness can lead to huddling behavior thus shedding light on the conundrum between aggregative behavior and evolutionary mechanisms.

Emperor penguins (*Aptenodytes forsteri*) are the only birds that breed during the Antarctic winter on fast-ice (sea ice that is “fastened” to the coastline) and are known to huddle (an active and close aggregation of animals) to survive extended stressful periods without food in the severe conditions of Antarctica winters [6]. Huddling allows penguins to minimize heat loss, lower their energy expenditure and reallocate the saved energy to other functions by thermoregulation [6]. During strong wind events, huddle density may be as high as 10 birds m^-2^ [4]. Penguins rotate positions constantly within the huddle, although the moving penguins remain together and the huddle as a whole appears semi-static [7]. However, due to the harsh conditions, observational study of the penguin huddling is difficult, and the specific dynamics of huddling behavior not well understood. Numerical models provide an alternative method to examine the dynamics and benefits of huddling behavior. Previous studies have used models to demonstrate how winds move around static huddles thus demonstrating the benefit of huddling as a mechanism to remain warm [4]. However, models of huddle formation that can assess the behaviors that lead to huddle formation require direct coupling because the fluid environment and penguin behavior both change in response to each other. We developed a coupled model that employs a finite difference scheme to compute the environmental conditions (wind field and ambient temperature), and a smoothed particle hydrodynamic model adapted to simulate penguin behavior. The models are coupled through the penguin positions, metabolic heat release, and local temperature allowing dynamic responses of both penguins and the ambient environment to each other.

During Antarctic winters, *A*. *forsteri* males are huddled on average 38% of the time [7] to maintain local ambient temperatures within their comfortable thermo-neutral zone (TNZ), varying from -10 to 20°C [6,8]. As penguins experience thermo-neutral environmental temperatures, they produce minimum and constant metabolic heat to maintain physiological activity at an optimal rate [6]. If ambient temperatures drop lower than the low critical temperature (LCT, -10°C), individual metabolic rates increase to maintain body temperature and individuals seek to form a huddle in order to minimize energy consumption [6]. By close packing in tight huddles, a penguins’ cold-exposed body surface is reduced greatly, and individual metabolic rates are depressed through reduction of individual heat loss to the environment [9]. Additionally, the combined heat loss of all huddling penguins increases local ambient temperature and helps stabilize huddles within this comfortable TNZ [9]. Penguin colony studies suggest that the average time spent by individuals in the interior of a huddle fluctuates around 50 minutes, and huddle durations are typically around 1.6h ± 1.7h [10]. When ambient temperatures are higher than the upper critical temperature (UCT, 20°C), metabolic rates increase as penguins move away from each other to cool down, and huddles disperse.

In recent years, huddling has been tested as a self-organizing system triggered by temperature [11–13], with each penguin relocating or remaining stationary to optimize its own metabolic activity [4]. Although some aggregate metrics of huddles (such as average internal temperatures) can be estimated by these mathematical models, the details describing motion and physical states of individual penguins provided by these models are limited. In addition, the constraints of existing models are very strict such as the huddle should maintain heterogeneity of shape to ensure penguins have equal access to warm center, but without providing details about how this equality is achieved [4,7]. To help understand the dynamics of penguin huddling, we coupled a traditional finite difference method (FDM) model for the wind and temperature field with an smoothed particle hydrodynamic (SPH) model adapted for penguin behavior. The coupling is two-way allowing the flow field to respond to changes in penguin position, and the penguins to respond to changes in the flow field and local temperature.

The FDM method is based on the application of a local Taylor expansion to approximate the non-linear differential fluid equations. FDM is comparatively easy to implement and has a relatively low computational cost. However, the FDM method is constrained by a fixed grid and individual particle properties are not maintained. In contrast, the SPH method is a mesh-free method that solves the Navier-Stokes equations from fluid mechanics. It uses a system of Lagrangian particles to represent a flow of bulk fluid through solution of the continuum hydrodynamic equations [14,15]. These hydro-particles carry their own physical quantities, such as mass, volume, temperature, velocity and density, and the interaction with other particles is defined by a kernel function. Gingold and Monaghan [16] and Lucy [17] simultaneously introduced SPH to solve astrophysical problems.

We modified an SPH model to represent animal behavior by incorporating specific terms. The advantage is that individual particle information is maintained, and each particle directly interacts with neighboring particles much as an animal. In the case of penguins, behavior can be implemented to make an individual search for a nearby penguin or group when local ambient temperature drops below LCT. As the penguin moves, the wind field responds to the moving obstacle. Therefore, a coupled method with FDM and SPH is established for penguin huddling simulation, where wind fields are solved with FDM and “penguins” are represented by SPH particles. These models are coupled through temperature (ambient, wind chill, and penguin internal) and position of penguins (as obstacles to wind flow) resulting in an algorithm that allows for two-way dynamic interactions between not only individual penguins, but also penguins and the ambient environment.

**Fig 1:**
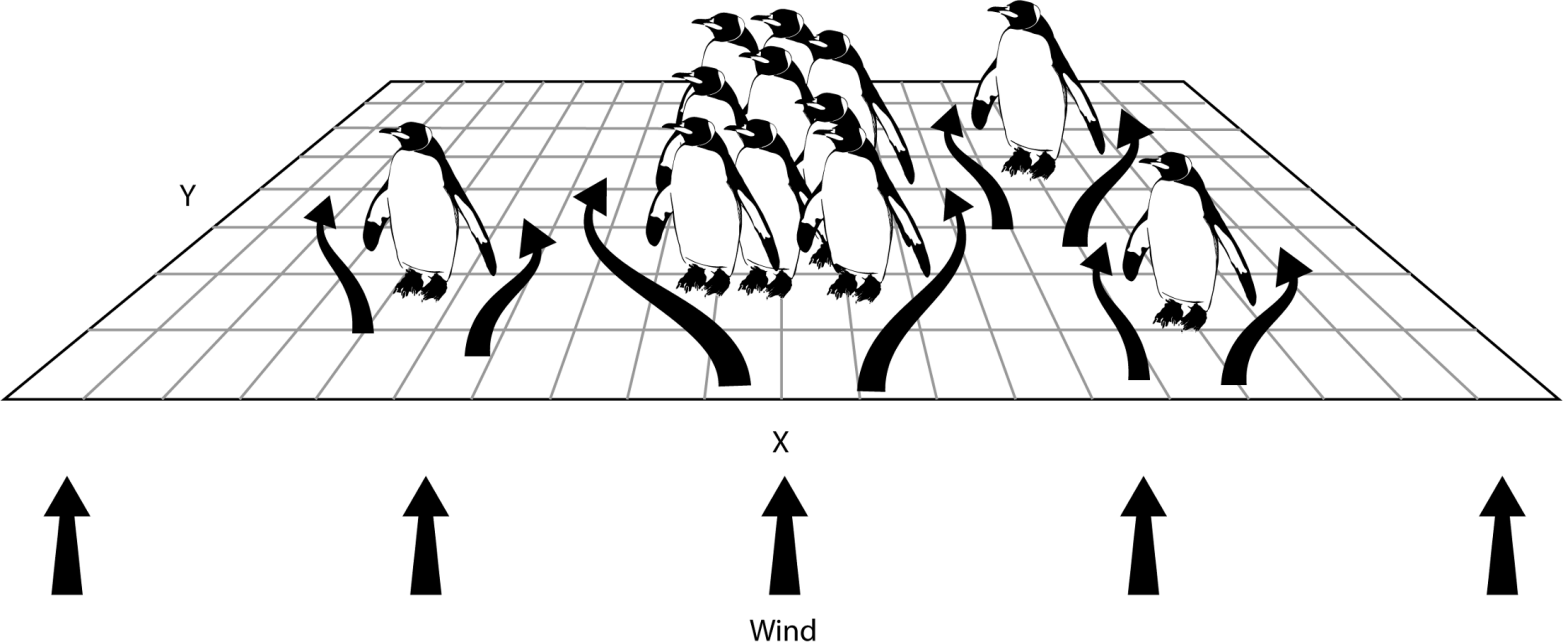
Model setting. Wind field is calculated using FDM on a finite 2D grid, where wind enters in the *x*-direction and interacts with penguins. Penguin behavior is simulated using SPH method. Flow responds to penguin movement within the grid.

Coupling of Eulerian and Lagrangian approaches in other engineering problems is a promising technique for studying complex fluid or coupled fluid-behavior systems [18–21]. This paper presents a new method of coupling finite difference methods (SPH-FDM) for estimating the wind field and smoothed particle hydrodynamics to simulate penguin huddling behavior (Fig 1). With this novel approach, each individual penguin’s state (i.e., position, velocity, temperature, metabolic heat loss, and path of motion) can be traced through implementation of the SPH method while solutions for wind velocity and direction across a complex and dynamic landscape can be easily quantified by FDM at a reduced computational cost. Section 2 describes the finite difference scheme for the wind and ambient temperature fields. Section 3 outlines the adaptation of SPH to model individual penguin behavior. Section 4 details the coupled method and implementation of the model. Section 5 reports results from a simulated penguin huddle. Section 6 highlights the advances provided by the model and outlines future applications and research directions.

## 2. Governing equations for wind field

The wind field is governed by the Navier-Stokes equations (NSE) (also see Appendix A):

$$\frac{\partial }{\partial t}u+u∙\nabla u=-\frac{1}{\rho }\nabla p+\mu \nabla^{2}u+\phi$$ (1)

∇∙u=0 (2)

Where ***u*** is the velocity vector of the fluid at a point, *∇* is the del operator defining spatial gradients, *ρ* is density, *p* is the fluid pressure, *μ* is the viscosity of the fluid, *∇*^2^ is the Laplacian operator, and ***ϕ*** consists of external body forcing terms. When resolving the wind field, (1) and (2) are solved at the current time, after which the velocity field is updated for the next time step. The Pressure Poisson equation (PPE) is used to solve the pressure term following [22]:

$$\nabla ∙\left(\frac{\partial }{\partial t}u+u∙\nabla u\right)=\nabla ∙(-\frac{1}{\rho }\nabla p+\mu \nabla^{2}u+\phi )$$ (3)

$$\nabla^{2}p=\nabla ∙(\phi -(u∙\nabla )∙u)$$ (4)

The movement and evolution of the wind are governed by the Navier-Stokes equations (NSE) of fluid motion in a continuum. The wind field simulation is only considered in two dimensions and can be expressed in the FDM as:

$$u_{t}+p_{x}=-(u^{2}{)}_{x}-(uv{)}_{y}+\gamma (u_{xx}+u_{yy})$$ (5)

$$v_{t}+p_{y}=-(v^{2}{)}_{y}-(uv{)}_{x}+\gamma (v_{xx}+v_{yy})$$ (6)

$$u_{x}+v_{y}=0$$ (7)

where subscripts refer to partial differentiation in the respective dimension. The two momentum Eqs 5 and 6 describe the time evolution of the wind velocity field under inertial and viscous forces, where *u* and *v* are flow velocity (m/s) in two directions, *u*_*x*_,*u*_*y*_,*v*_*x*_,*v*_*y*_ are spatial gradients of *u* and *v* in the two directions; *u*_*xx*_,*u*_*yy*_,*v*_*xx*_,*v*_*yy*_ are divergences of the spatial gradient in the two directions; and *p*_*x*_,*p*_*y*_ are gradients of pressure (Pa) in two directions.

The Pressure Poisson equation (PPE) is used to solve the pressure term [22]. At time *n*+1 (the pressure that corresponds with the velocity at *n*+1)

$$u^{n+1}=u^{n}+\Delta t(-u^{n}∙\nabla u^{n}-\frac{1}{\rho }\nabla p^{n+1}+\mu \nabla^{2}u^{n})$$ (8)

$${\nabla ∙u}^{n+1}={\nabla ∙u}^{n}+\Delta t(-\nabla ∙(u^{n}∙\nabla u^{n})-\frac{1}{\rho }\nabla^{2}p^{n+1}+\mu \nabla^{2}{(\nabla ∙u}^{n}))$$ (9)

Forcing ∇ ∙ ***u***^*n*+1^ = 0 to satisfy continuity, the Poisson equation for *p* at time *n*+1 is then:

$$\nabla^{2}p^{n+1}=\rho \frac{\nabla ∙u^{n}}{\Delta t}-\rho \nabla ∙(u^{n}∙\nabla u^{n})+\mu \nabla^{2}(\nabla ∙u^{n})$$ (10)

The velocity field is then updated using the pressure correction before proceeding to the next time step.

## 3. Penguin behavior simulation using SPH

**Fig 2:**
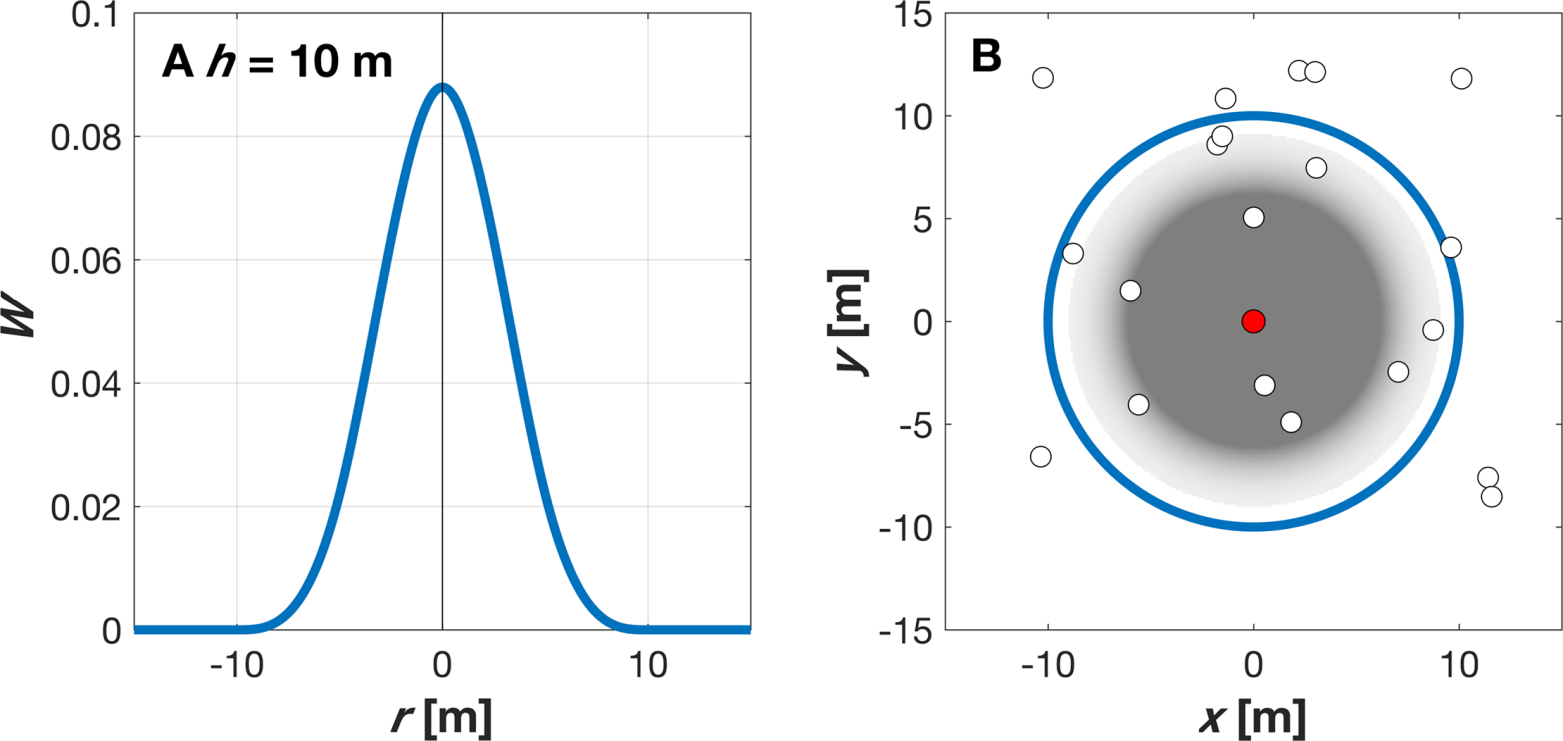
Kernel mapping for SPH model. (a) Smooth kernel value depends on distance from individual, and (b) only particles (open circles) within smooth length of the particle (red circle) contribute because *W* falls off rapidly for *r* ≥ *h* and the interactions are zero for *r* > 2*h*.

In the SPH method, the fluid field is discretized and solved by a series of particles representing individual penguins. Using this method, the interpolated value of a particle parameter (*F*_*part*_*)* at any position (***r****)* can be expressed as [16]:

$$F_{part}(r)=\int F_{part}(r\prime )W(r-r^{\prime },h)dr^{\prime }$$ (11)

where the integration is over the entire continuous domain, (*h*) is a smoothing length, and (*W*) is a weight function called the interpolation kernel. For the simulation model, the integral interpolant in (11) is approximated by a summation as:

$$F_{part}(r)=\sum_{i}m_{i}\frac{{F_{part}}_{i}}{\rho_{i}}W(r-r_{i},h)$$ (12)

where *m*_*i*_ and *ρ*_*i*_ are the mass and density of particle *i* and the sum is over all particles. In this application, the smoothing kernel *W(r*, *h)* is specified to be a *C*^*2*^ spline based interpolation (a special type of piecewise polynomial) with radius *2h*, which approximates the shape of a Gaussian function (Fig 2):

$$W(r,h)=\frac{7\pi }{10h^{2}}\left\{ \begin{array}{l} 1-\frac{3}{2}q^{2}+\frac{3}{4}q^{3}\;if\;0\leq q\leq 1\\ \frac{1}{4}(2-q{)}^{3}\;if\;1\leq q\leq 2\\ \;0\;otherwise \end{array}\right.$$ (13)

where $q=\frac{r}{h}$ and *h* = 10 m.

The spatial gradient of the function (*F*_*part*_) is given by differentiating the interpolation equation as [16]:

$$\nabla F_{part}(r)=\sum_{i}m_{i}\frac{{F_{part}}_{i}}{\rho_{i}}\nabla W(r-r_{i},h)$$ (14)

The mass conservation equations for penguin huddle model are given as:

$$\rho_{j}=\sum_{i}m_{i}W_{ji}$$ (15)

$$\frac{d\rho_{j}}{dt}=\sum_{i}m_{i}(v_{i}-v_{j})\nabla W_{ji}$$ (16)

where *ρ*_*j*_ is the density of particle *j* with velocity *v*_*j*_, and *m*_*i*_ is the mass of particle *i*. The position vector ***r*** from particle *i* to particle *j* is calculated by ***r***_***ji***_ = ***r***_***j***_−***r***_***i***_. *W*_*ji*_ = *W*(*r*_*ji*_,*h*) is the interpolation kernel with smoothing length *h* evaluated at a distance |*r*_*ji*_|.

The momentum equation for particle, *j*, is:

$$\frac{Dv_{j}}{Dt}=-\sum_{i}m_{i}(\frac{P_{i}}{\rho_{i}^{2}}+\frac{P_{j}}{\rho_{j}^{2}})\nabla_{j}W_{ji}$$ (17)

The contribution force from particle *i* to particle *a* when the kernel is a Gaussian is:

$$F=\frac{2m_{j}m_{i}}{h^{2}}(\frac{P_{i}}{\rho_{i}^{2}}+\frac{P_{j}}{\rho_{j}^{2}})(r_{j}-r_{i})W_{ji}$$ (18)

Finally, penguin particles are moved throughout the simulation using:

$$\frac{{dr}_{j}}{dt}=v_{j}$$ (19)

The movement (*v*_*j*_) of an individual penguin is calculated using a modified form of the Navier-Stokes equation adapted for animal behavior:

$$\frac{\partial v_{j}}{\partial t}-b_{j}+\frac{\nabla P_{j}}{\rho_{j}}=0$$ (20)

where ***b*** is an acceleration term (m/s^2^) that compels “uncomfortable” penguins (i.e., those that are above or below their TNZ) to relocate their current positions, and *p* is a repulsive pressure between individual penguins that prevents them from overlapping neighbors. The repulsive pressure between one particle and its neighbors is driven by the SPH momentum Eq (17). In the SPH behavior model, the advection term (i.e. ***v*** ∙ ∇***v***) is not included as there is no acceleration of penguins deu to the flow of penguins, nor is the dispersion term needed (i.e. *μ*∇^2^***v***) because the penguins are not dispersed due to gradients in the flow. However, the dispersive term could be included at a later point to simulate a level of random behavior associated with other environmental cues.

The body acceleration parameter ***b*** is a function of the temperature that an individual penguin experiences or perceives. This experienced temperature is in turn dependent on the ambient temperature, the cumulative metabolic heat loss of the individual and near neighbors, and the wind chill. Ambient temperature and wind velocity are stored at each grid point in the FDM; therefore, ambient temperature, *T*_*a*_, and wind velocity magnitude, *u*, are calculated by taking average of temperatures and wind velocities from the grid points immediately adjacent to each penguin and provided to the SPH penguin behavior model.

**Fig 3:**
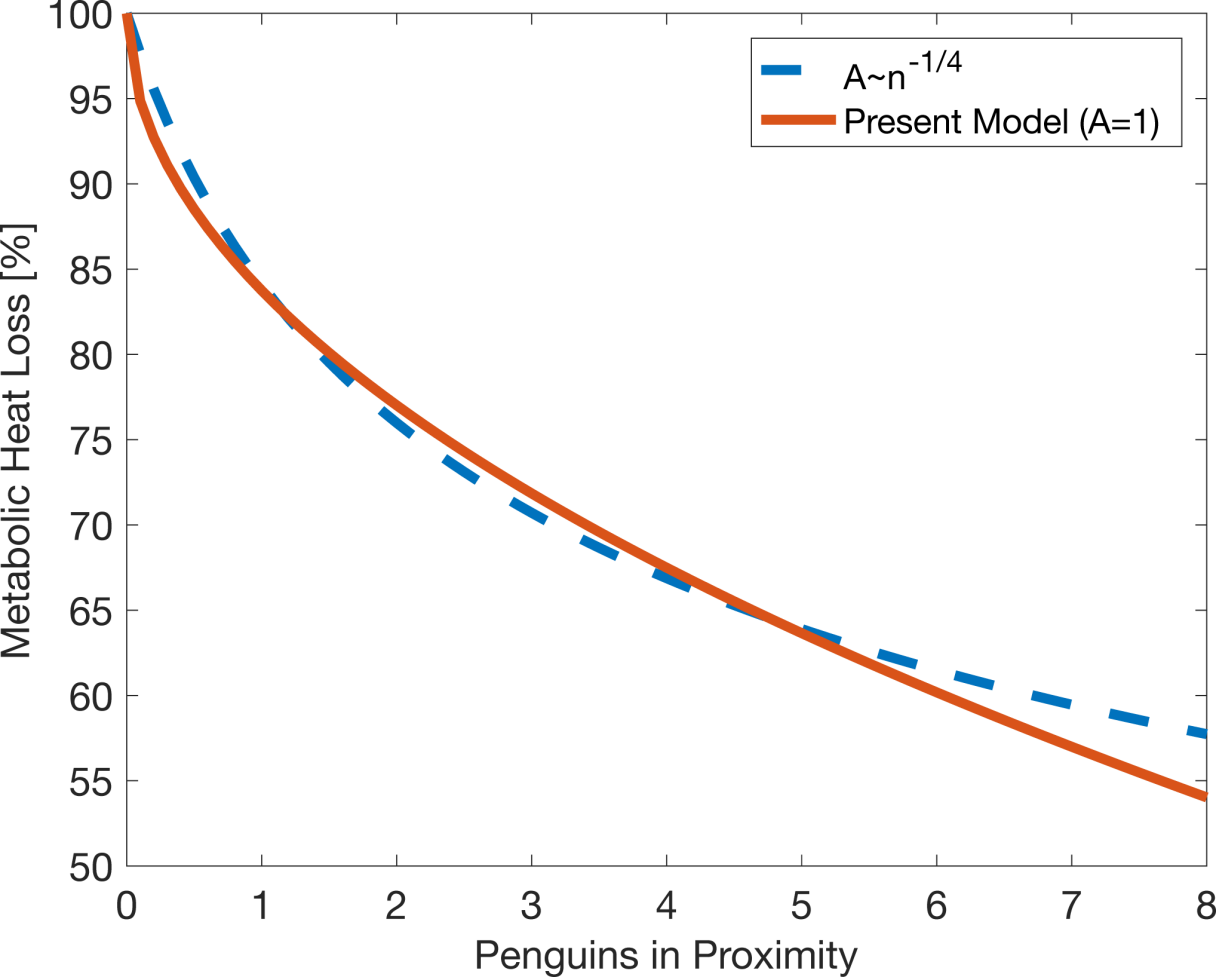
Comparison of metabolic heat loss formulations. Blue line uses (22) with *A* proportional to number of penguins in proximity. Red line (our model) updates *Tw* dynamically and *A* is constant. Metabolic Heat Loss is the percentage relative to the heat lost for a single individual.

Individual penguins produce and release metabolic heat through their cold-exposed body surface. Metabolic heat production is calculated based on Fourier’s Law of heat flow [6]:

$$MR=CA(T_{b}-T_{a})$$ (21)

where *MR* is metabolic heat production (in Watts, W), *C* is whole body thermal conductance of a penguin (0.104 W°C^-1^) [23], and *T*_*b*_ is a constant individual body temperature (37.7°C). Previous models on mammal huddling suggest that the reduced proportion of an animal’s surface area, *A*, depends on the number *n* of aggregated animals [11–13], which can be calculated as:

$$A=n^{-\frac{1}{4}}$$ (22)

where *n* is the number of huddling individuals. However, we do not include this form because we are directly estimating the wind velocity around the animal and the wind speed and ambient temperature change dynamically. An individual that is close to another penguin will thus experience a reduced wind velocity and warmer ambient temperature. Therefore, the effects of individual penguin proximity are implicit in our model and *A* is set to unity. Comparison of the formulation in (22) with our formulation where *A* is constant and *T*_*wc*_ changes shows almost identical relationships with the number of penguins around an individual (Fig 3).

In the model, the released metabolic heat warms up the local air and increases the temperature in the vicinity of the penguin. The increased temperature due to released metabolic heat is calculated as:

$$\Delta T=\frac{\sum_{d}{MR}_{i}\frac{{F_{part}}_{i}}{\rho_{i}}W(r-r_{i},h)}{C_{air}\rho_{air}V_{air}}$$ (23)

where *C*_*air*_ is the specific heat of air (1000 kJ kg^-1^°C^-1^ at -50°C), *ρ*_*air*_ is the density of air (1.4224 kg m^-3^ at -25°C), and *V*_*air*_ is the volume of a smooth cylinder with diameter *h* and a length of an average penguin height (1.2 m). The temperature in the vicinity of each penguin, *T*_*exp*_, is then updated as:

$$T_{exp}=T_{a}+\Delta T$$ (24)

Wind flow around and through the huddle affects individual penguins differently through the wind-chill effect depending on the wind velocity and the exposure of individual penguins [4]. In this study, wind chill for each particle penguin is calculated by a standard North American and United Kingdom wind chill index equation:

$$T_{wc}=\left\{ \begin{array}{cc} 13.12+0.6215T_{exp}-11.37u^{0.16}+0.3965T_{exp}u^{0.16} & for\;u>1.3\;m\;s^{-1}\\ T_{exp} & for\;u\leq 1.3\;m\;s^{-1} \end{array}\right.$$ (25)

where *T*_*wc*_ is the wind chill temperature (°C) felt by each computational particle. In this wind chill equation, the calm wind threshold is around 1.3 m s^-1^, and there is no effect if wind speeds are under this value [24]. The penguin exposed temperature, *T*_*exp*_, is then corrected for wind chill to *T*_*wc*_ to determine if individual particles are within their TNZ.

**Fig 4:**
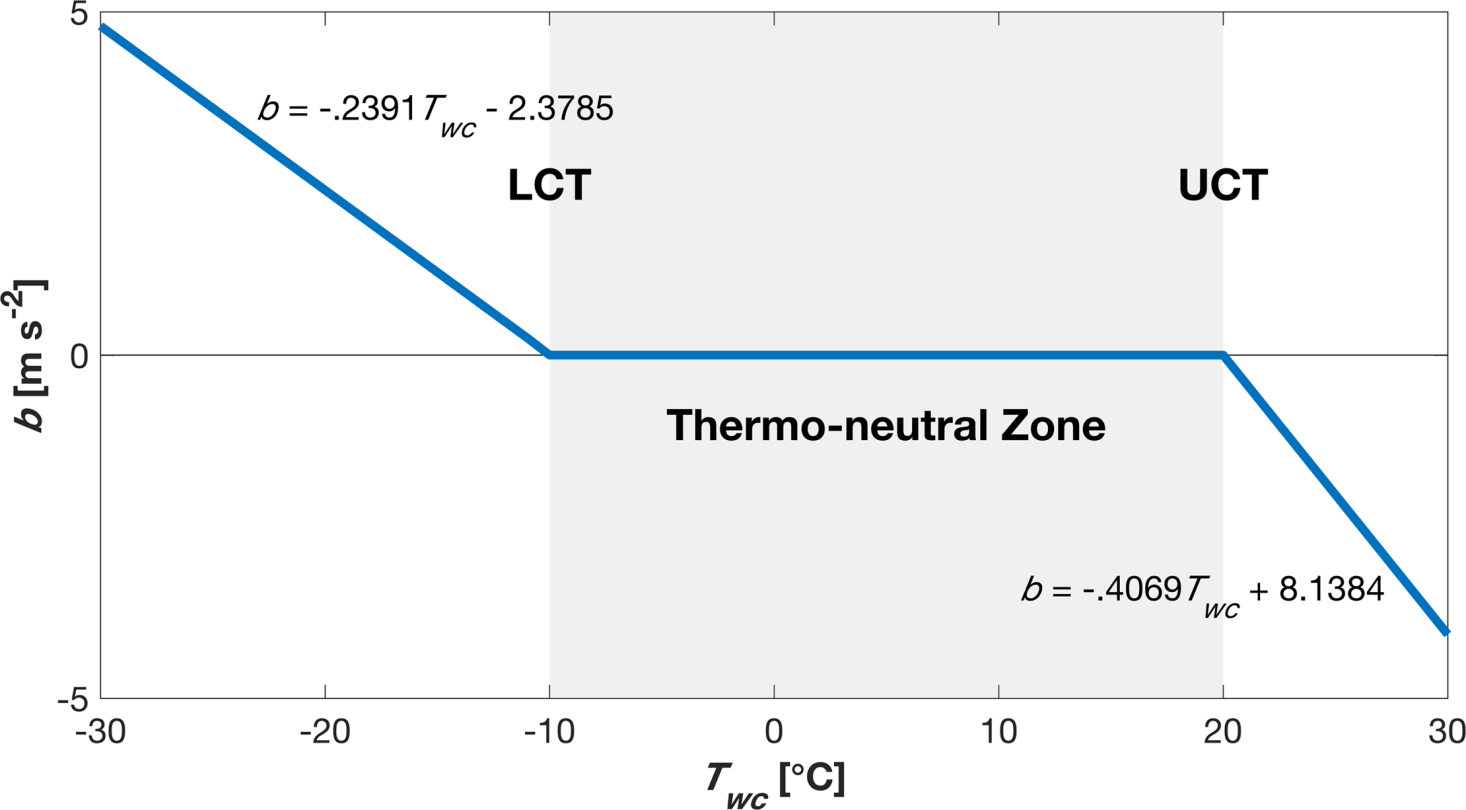
Relationship between exposed (wind chill) temperature (*T*_*wc*_) and penguin acceleration (*b*). Individual penguins will move towards the huddle (*+b*) if *T*_*wc*_ < LCT or away from the huddle (*-b*) if *T*_*wc*_ > UCT.

The functional dependence of ***b*** on *T*_*wc*_ (Fig 4) is set so that “cold” penguin particles move towards the huddle center of mass (i.e., to warmer areas) and “hot” penguins away from the huddle center (i.e. to cooler areas). ***b*** represents the individual motivation to remain within a comfortable thermal zone, which in this case, would be to move closer to other penguins thus receiving the benefit of other’s metabolic heat loss and reducing wind exposure. The functional relationship (Fig 4) is further set so that individual penguins move at a maximum speed of ~0.5 m s^-1^ similar to observed land movement speeds [25]. Initially, for a randomly distributed group of animals, an individual would move towards the nearest conspecific, but as groups form, animals would opt to move towards the perceived largest group because more animals would increase heat gain form others and reduce wind exposure.

For now, we set *b* so that penguins move toward the center of mass of the penguin distribution. Because stressed penguins will likely move to the nearest penguin or the largest group of penguins to find a comfortable place to stay warm, huddle formation will occur at random locations, or multiple huddles may form due to inaccurate perceptions of huddle size. Prescribing a single huddle formation at the domain center prevents potential issues of huddle formation near domain boundaries, which can cause numerical issues. Modification of the model to allow for random locations and multiple huddles is a planned future improvement to the model. For the purposes of this study, to address whether individual based motives lead to huddle formation, the specific location of a huddle, or the number of huddles does not affect results.

Several functional forms between ambient temperature and magnitude of parameter ***b*** were evaluated, and a linear relation was chosen for this study as results did not vary significantly between forms of the parameterization. When penguin exposed temperature *T*_*wc*_ increases from very low temperatures, the *b* parameter diminishes to zero at the LCT (-10°C; Fig 4). As *T*_*w*_ increases further, *b* becomes negative at the UCT (20°C) and accelerates particles away from the colony’s center.

This implementation of penguin responses to changes in temperature mimics huddling behavior through an algorithm that minimizes heat lost for individual penguins. In other words, the model considers huddling not as an empathetic behavior where penguins cooperate for the good of the colony, but as an individual strategy to minimize energy expenditures with no knowledge or concern of the state of neighbors.

## 4. FDM-SPH coupling and implementation

Penguin particles move in response to ambient temperature as determined by the individual body temperature, the aggregate heat given off by neighboring penguins, the effects of wind chill, and the background ambient environmental temperature. As the trajectory of a particle is computed, the quantities of heat release and momentum gained or lost by all particles are incorporated in subsequent calculations. For SPH-FDM coupled method, the penguin positions and resultant ambient temperature are the primary parameters exchanged by these coupled methods.

Individual penguins are initially randomly located on a flat plane with dimensions of 50 m x 100 m with no obstacles impeding penguin movement other than other penguins (Fig 1). Three simulations matching the size of huddles during distinct periods of pairing (~200 penguins), incubation (~800 penguins), and chick rearing (~100 penguins) are used in the simulations [10]. Penguins interact with the wind field according to their distribution across the finite grid. Wind enters from the upstream *y*-direction boundary in this application where it interacts with, and is impeded by, the distribution of penguins across the field’s computational grid. Wind speed and ambient temperature then determine the heat loss for individual penguins.

**Fig 5:**
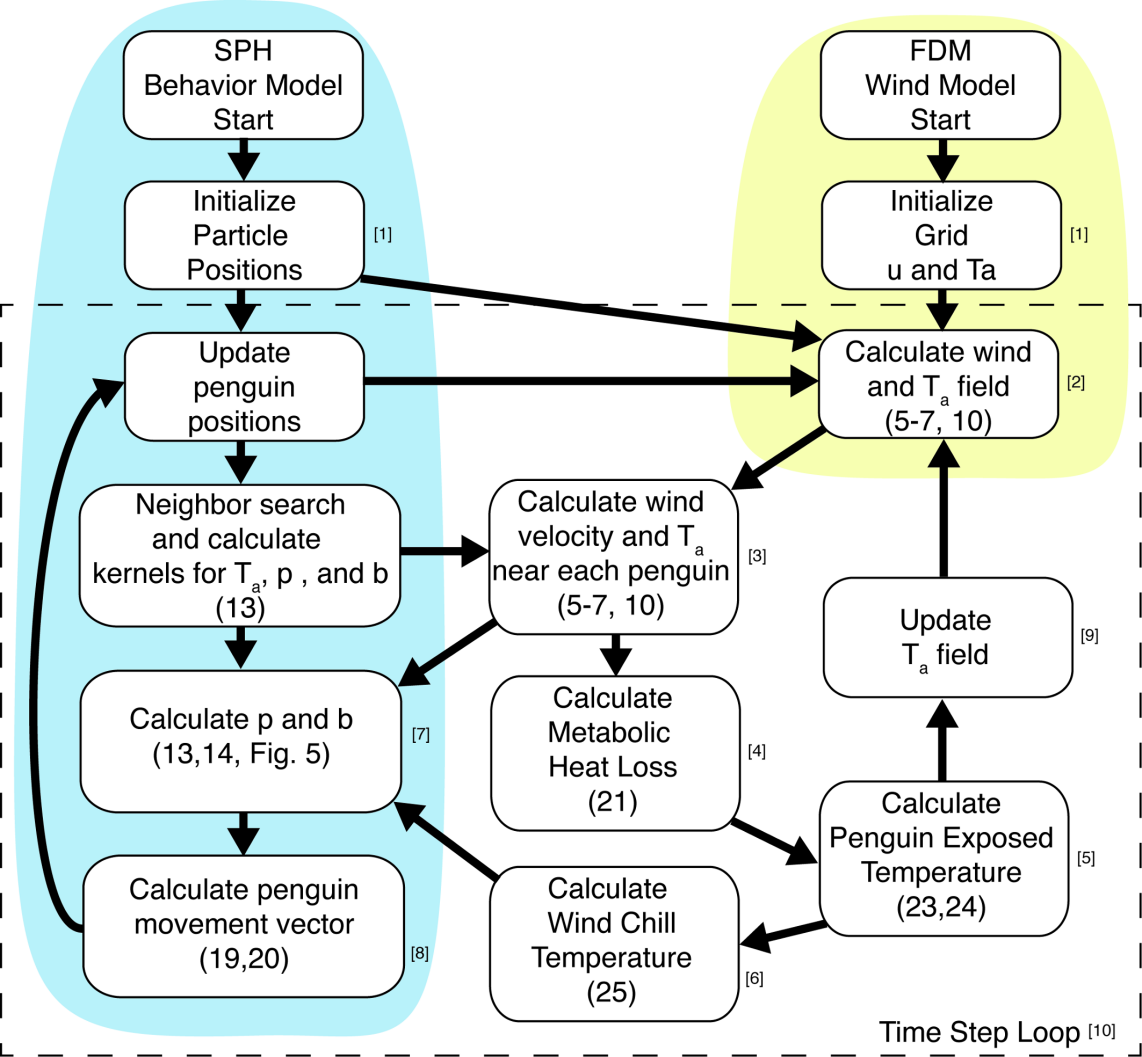
Flowchart for the coupled fluid-behavior model. Wind field is computed using the FDM method (red shading) then provides wind velocity and ambient temperature data to the behavior model. Penguin behavior is then calculated using the SPH method (blue shading) and provides new positions and ambient temperatures around individual penguins back to the wind model. Unshaded portion indicates where model is coupled. Numbers in brackets correspond to the steps outline in the text.

The average radius of a penguin “particle” is assumed to be approximately 0.25 m. As the particle size and grid size should be the same magnitude to reduce truncation error and maintain stability of the numeric models [24], a similar grid size of 0.25 m is chosen to couple the SPH model and FDM model. Due to the small grid size and target wind velocities, a small time-step of 0.02 seconds is required to prevent numeric oscillation and divergence. Because the time step is so small relative to penguin movements, the wind velocity is updated every 30 seconds (simulation time) as individual penguins move and the huddle shape forms, deforms or disperses. The procedure to simulate huddle formation is as follows (Fig 5):

1. At initial time step *t*_*o*_, randomly locate penguins within domain and initialize FDM grid with initial velocity and pressure.
2. Compute the wind velocity and ambient temperature field using the FDM wind model.
3. Compute ambient temperature and wind velocity around each penguin for the SPH behavior model.
4. Compute individual metabolic heat release MR for each penguin.
5. Compute local temperature increase Δ*T* due to metabolic heat loss and update penguin exposed temperature *T*_*exp*_ by adding ambient temperature *T*_*a*_ and Δ*T*.
6. Correct penguin exposed temperature *T*_*exp*_ to account for wind chill and update to *T*_*wc*_.
7. Calculate *p* and *b* using *T*_*wc*_ (Fig 5).
8. Calculate penguin velocity vector using SPH behavior model.
9. Substitute *T*_*a*_ with *T*_*exp*_ in (6) and update penguin positions for next time step in FDM wind model.
10. Repeat steps 2–8 for next time step until simulation completes.

## 5. Results and discussion

**Fig 6:**
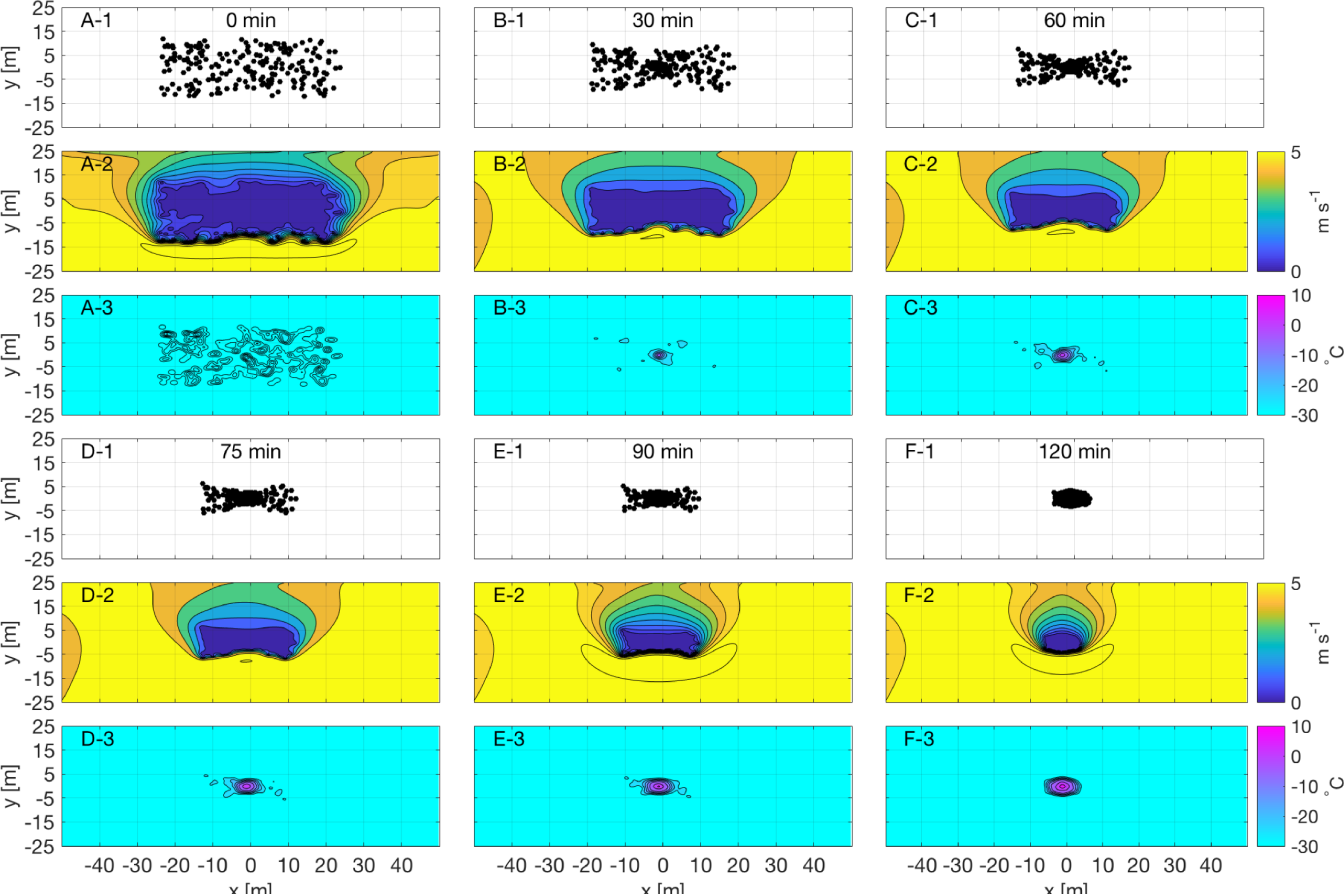
Penguin huddle formation over 2-hour simulation. (A-1) pengion positions, (A-2) wind velocity field [m s^-1^], and (A-3) wind chill temperature [°C] at time *t*_*o*_ (A), (B-F) same as in (A) but for 30 min, 60 min, 75 min, 90 min and 120 min since the start of the simulation.

The proposed coupled model is applied to the simulation of penguin huddling, and the coupling technique is validated by comparison to field observations of huddle formation. Huddle formation is observed in the model over the 2-hour simulation. As a huddle forms (Fig 6; upper panel), the cumulative metabolic heat released from penguins in a neighborhood accumulates, and the tight aggregation of penguins blocks wind from flowing around individuals (Fig 6 middle panel)–thereby elevating local ambient temperature and eliminating wind chill (Fig 6 lower panel; Table 1). When ambient temperatures fall within the TNZ (Typically -10 to 20°C), penguins are less motivated to move, and the huddle becomes stable. Throughout the simulation, the positions of individual penguin can vary as neighbors push each other vying for an optimal location within the huddle.

**Table 1:** Summary of descriptive statistics. Mean exposted temperature, range, percent of penguins in the thermo-neutral zone, and huddle density for each fo the time periods presented in Fig 6.

Fig 6 Panel	Time (min)	Penguin exposed Temperature (°C ± σ)	Penguin exposed Temperature Range (°C)	% of Penguins in TNZ	Average Huddle Density (penguins/m^2^)
A	0	-26.97 ± 0.92	-28.00 ~ -24.09	0	0.2
B	30	-23.78 ± 6.07	-27.98 ~ -2.05	5.5	0.3
C	60	-18.38 ± 9.37	-27.97 ~ 2.42	25.5	0.4
D	75	-14.87 ± 10.00	-27.88 ~ 3.80	37.5	0.7
E	90	-11.42 ± 10.01	-27.67 ~ 5.56	53.5	1.2
F	120	-5.03 ± 6.85	-23.12 ~ 7.74	77.5	3.1

**Fig 7:**
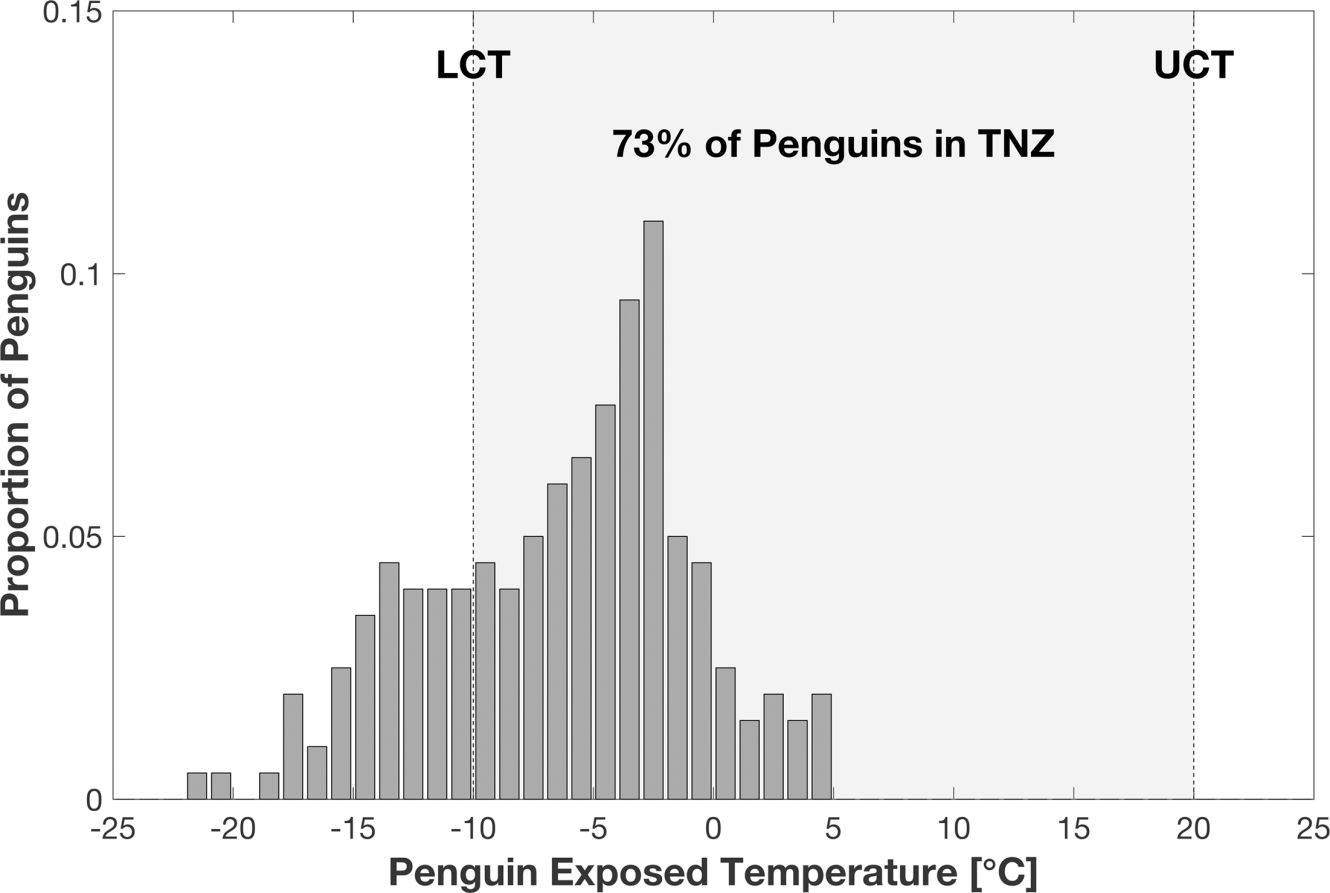
Distribution of penguin exposed temperature at end of simulation (2 hours).

**Fig 8:**
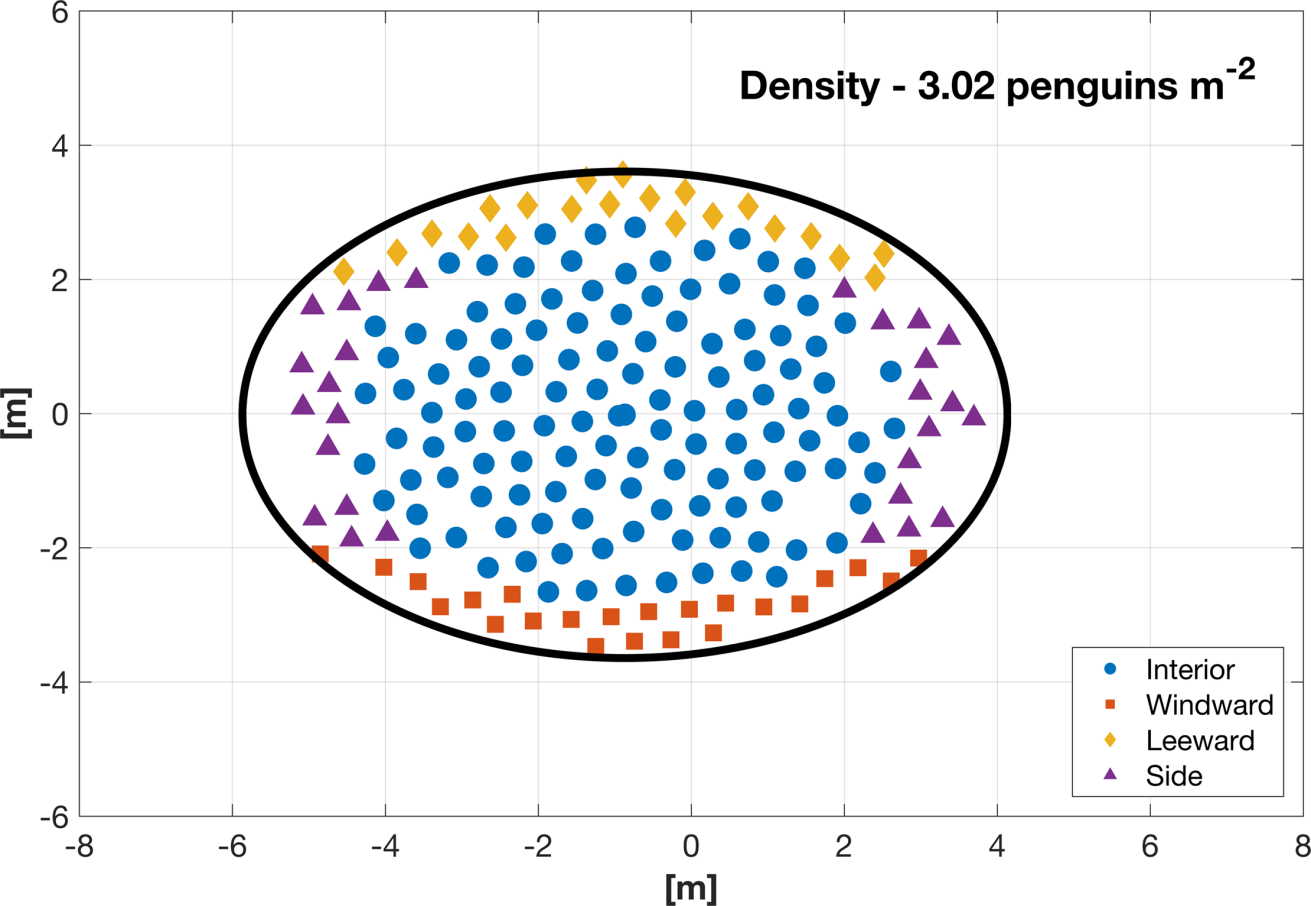
Huddle density and dimensions. Black circle shows the estimated huddle size used for density computations. Symbols indicate whether an individual was on the windward (red square), leeward (yellow diamond), side (purple triangle), or interior (blue circle) of the huddle. Symbols are same for Figs 8 and 9.

Individual penguins relocate from loose aggregations to a stable and dense huddle until the interior penguin exposed temperature reached around 5°C. After the 2-hour simulation, approximately 75% of the simulated penguins are in their TNZ (Fig 7). The simulation model forms a huddle around 9×7.25 m (area ~ 51 m^2^; Fig 8). With 200 penguins, the density of huddle is around 3.02 birds m^-2^, which is comparable to the typical mean density of 2.8 birds m^-2^ shown in emperor penguin huddles during winter [8]. While the movement of the aggregated huddle may also be affected by wind direction, the ambient temperature is the main meteorological factor influencing huddling group density. In this simulation model, the body force term is determined only by ambient temperature and wind chill. However it is likely that a change in wind direction will not affect huddle formation, but simply shift the progression of the huddle as the location of the windward and leeward faces change in response to the wind direction change.

**Fig 9:**
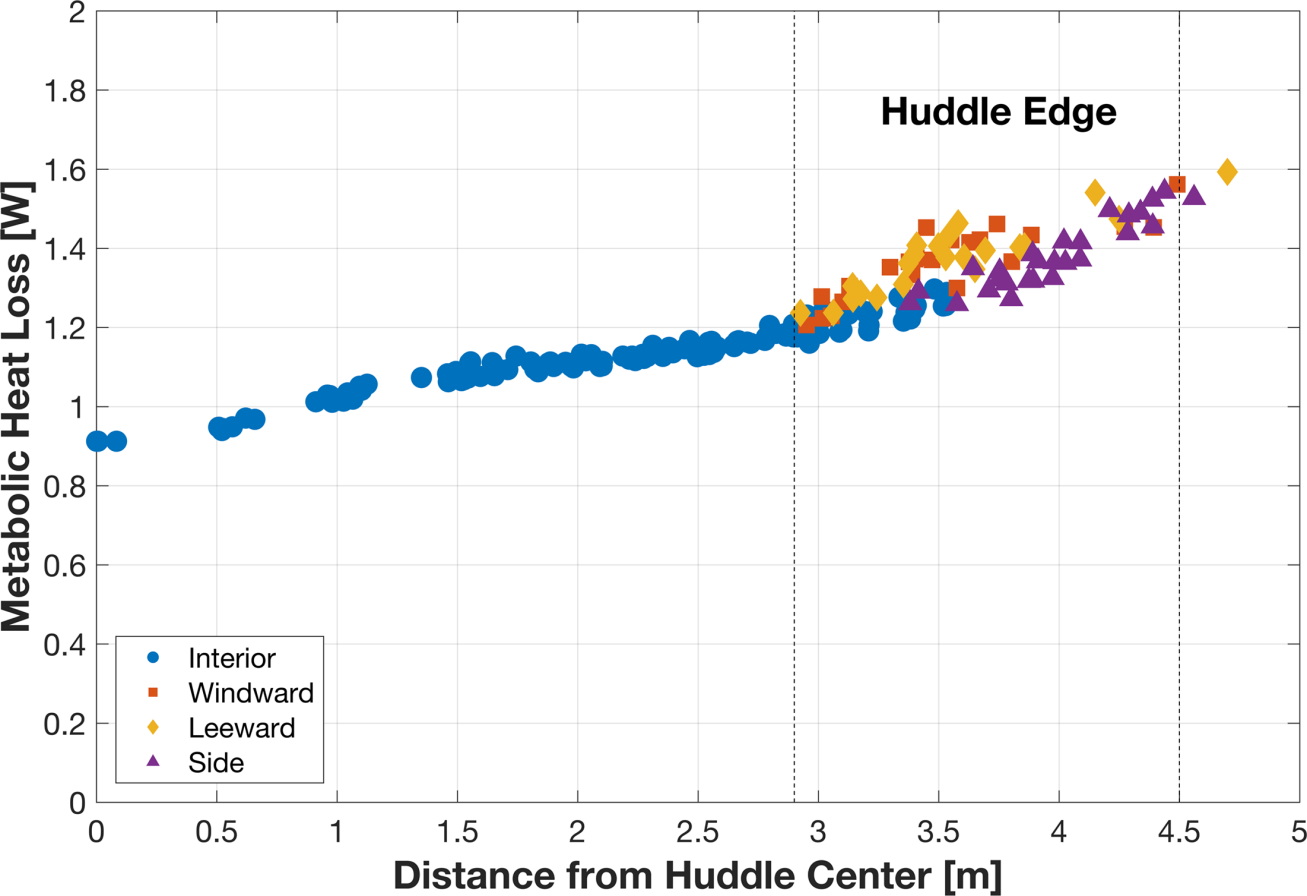
Relationship between individual metabolic heat loss and its distance to huddle center. Metabolic heat loss for penguins on windward, leeward, side, or interior of the huddle. Dashed vertical lines indicate the huddle edge.

**Fig 10:**
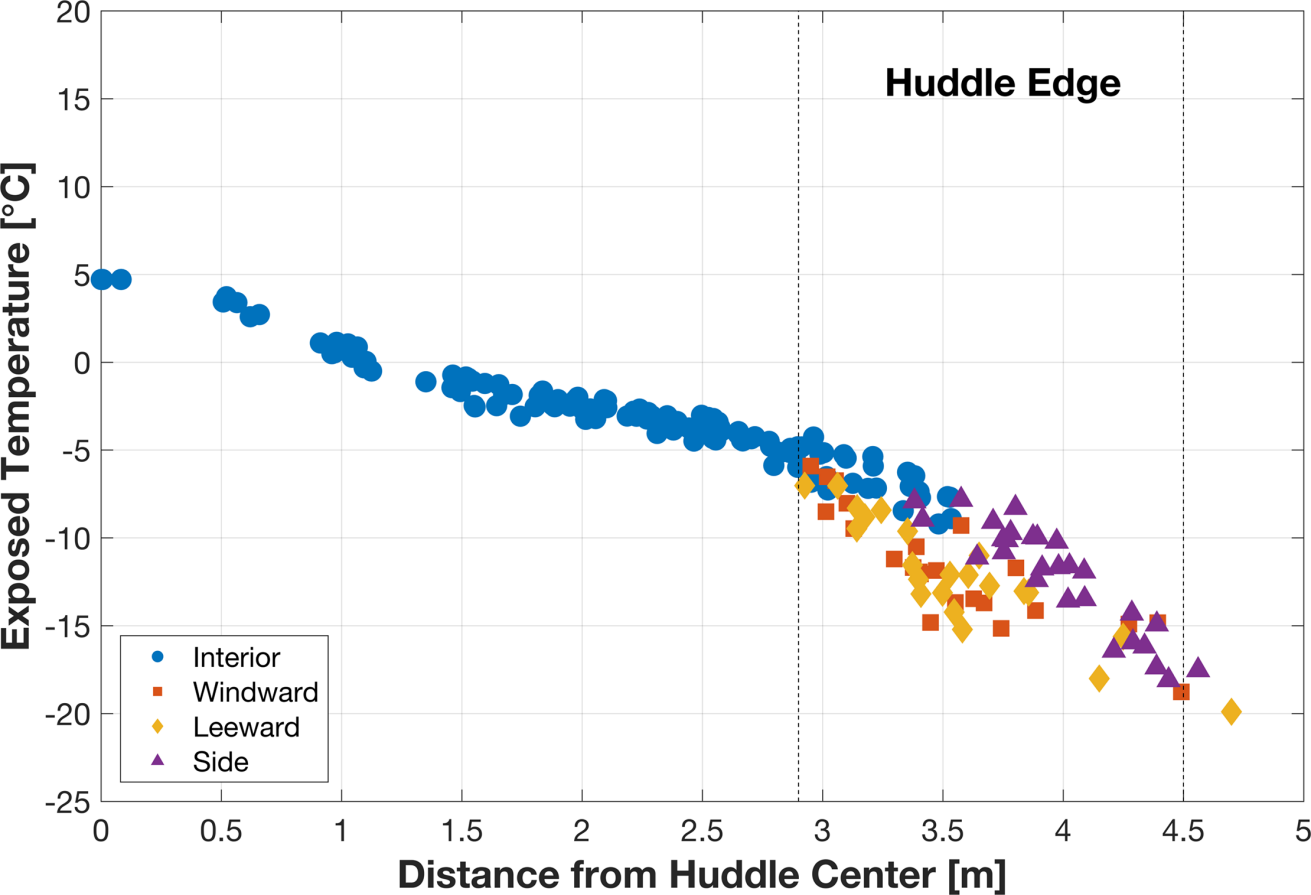
Relationship between individual penguin exposed temperature and distance to huddle center. Exposed temperature for penguins on windward, leeward, side, or interior of the huddle. Dashed vertical lines indicate the huddle edge.

Penguins on the huddle perimeter (Fig 8) release more heat than those in the interior because of a higher temperature gradient and proximal exposure to wind and associated wind chill effects (Figs 9 and 10). Because the ambient temperature of penguins around the perimeter was not in TNZ, these penguins continue to seek a more comfortable position within the huddle and were not stationary, but because other penguins block movement into the interior, they tend to move around the circumference of the huddle. Penguins on the perimeter windward facing side of the huddle experience lower temperatures because of the wind chill effect, which motivates them to move to a warmer position, such as a leeward or interior position. This simulation result supports observations that penguins, which are most exposed to the wind, move along the opposite flank of the huddle for protection [7,10].

In this simulation model of 200 penguin particles, ambient temperature and wind speed are the main factors affecting penguin huddles. At the beginning, penguins are affected by both ambient temperature and wind speed (Fig 6). After huddle formation, wind chill affects only penguins on the perimeter of the huddle. Exposed temperature and wind speed are good predictors of huddling occurrence in agreement with previous observations [8].

**Fig 11:**
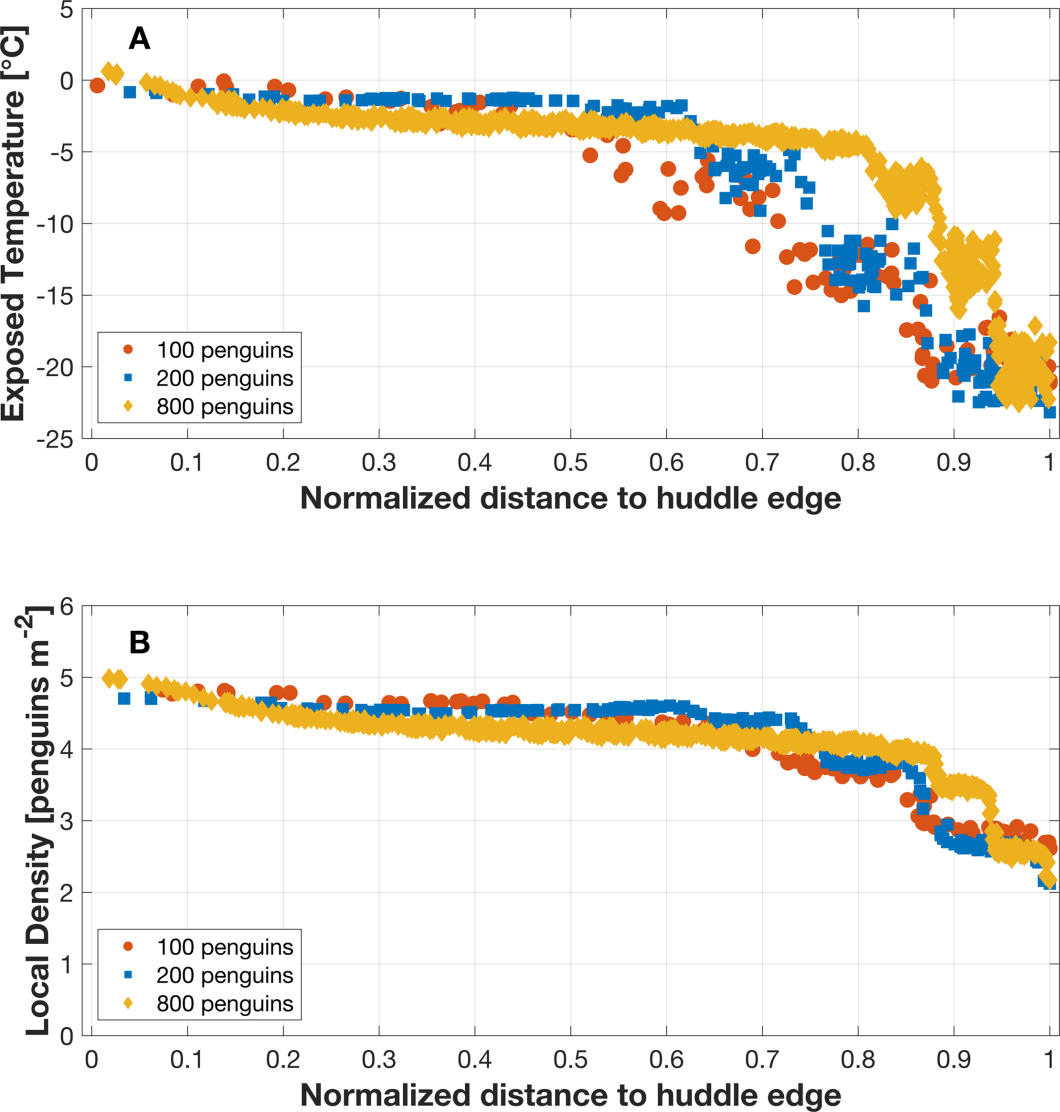
Relationship between percent of huddle radius in three sizes of huddles. (a) Penguin exposed temperature, and (b) local penguin density within the huddle for 100, 200, or 800 penguins normalized by the huddle diameter.

The breeding stages (pairing ~200 penguins, chick-rearing ~100 penguins, and incubation ~800 penguins) affect the number of huddles and the number of individuals per huddle [10]. When air temperatures decrease, large huddles are more frequent. Less dense huddles are more frequent during chick-rearing stage compared to other periods. These three penguin huddle sizes (100, 200, 800 penguins) show similar trends, where the highest density and highest ambient temperature are the center of the huddle (Fig 11A). Penguins on the boundaries are clearly affected by wind chill for all three-huddle sizes, but the density and temperature inside each huddle size is different for each for a given wind speed and ambient temperature. The trend of penguin-exposed temperature is similar to the trend in the local number density (Fig 11B). In the SPH calculation, particles within the smooth circle contribute to the temperature of the center particle as quantified by the kernel function. The more penguins inside the smooth circle, the more released metabolic heat is contributed to the center particle. The 800-penguin huddle shows approximately a linear decrease in penguin-exposed temperature along the huddle radius, and around 85% of penguins are within their TNZ. The curve decreases rapidly past the huddle perimeter due to wind chill. Around 70% of penguins remain in their TNZ in the 200-penguin huddle and around 60% of penguins remain in their TNZ in the 100-penguin huddle. Comparing the results of three huddle sizes, large huddles may keep more penguins remain in their TNZ providing insight into why large huddles are more frequent when air temperature is low.

## 6. Conclusion and future applications

In this study, a coupled fluid-behavior numerical model was developed to simulate the penguin behavior (SPH) in a dynamic wind field (FDM). Huddle formation is controlled by an individual penguin’s desire to remain in the TNZ and minimize heat loss [4,6,12], and not by a cooperative behavior aimed for the good of the colony. However, because the behavior is mutually beneficial, the individual behavior results in colony benefit. In the simulated huddles, most of the interior penguins reached a thermo-neutral temperature. For the penguins on the huddle perimeter, the ambient temperature was still below the thermo-neutral zone, which motivates those penguins to move toward more comfortable locations within the huddle (either in the interior or around to the rear of the huddle). Presumably as penguins on the edges (coldest exposed penguins; Figs 8 and 9) move towards the leeward side, new interior penguins will be exposed and exhibit similar behavior resulting in a steady state where the huddle is continually turning over and all penguins achieve a significant amount of time within their TNZ [7]. The huddle then forms a semi-static traveling wave as penguins continually reposition around the edge. Traveling waves have been hypothesized as either a response to a single penguin movement [26], or a form of communal behavior allowing all penguins to have some amount of time in the huddle center [7]. However we show that such waves occur when penguins seek only to maximize their own individual fitness.

Penguin huddling behavior represents one end of a spectrum of animal aggregation behavior where individual drive to maximize fitness is synergistic with the group benefit. Forming a huddle maximizes individual benefit and equally benefits the colony providing a clear explanation of how this behavior developed evolutionarily, and also likely leads to wave development in dynamic huddles as individuals seek optimal positions. The trade-off between individual and colony benefit may likely determine the maximal observed huddle sizes during different life stages (mating, incubation, and chick-rearing) where other behaviors may be more beneficial for individuals [10], or such sizes could simply be the result of temperature variance over the season (mating in early winter, incubation over winter, and chick rearing in late winter) as larger huddles are more common during colder weather. Our model at least partially supports the latter hypothesis; however, such questions could be explored in future modeling efforts coupled with experimental or observational study.

Future applications of this model will consider the individual penguin motion within a formed penguin huddle to determine how long cold-exposed penguins remain on the huddle perimeter before finding shelter within the interior (as well as the mechanisms that lets that happen), and how long individual penguins remain within the TNZ during a storm event. Another will examine the dynamics of huddle breakup as ambient temperatures rise and the huddle is no longer needed. Besides wind speed and ambient temperature, other environmental factors, such as solar radiation, wind direction and relative humidity can influence on huddle patterns [10]. These factors will be considered in the future and will be added to the penguin behavior model.

Our coupled model also has widespread applicability across disciplines. The model could be applied to other interesting ecological systems with fluid-fluid properties such as fish shoaling in river currents, fish schooling behavior, large mammal herding behavior, or bees swarming in a wind field. The advantage of the SPH method introduced here for aggregative behaviors such as fish schooling is that the direct interactions of an animal with its neighbors is implicit in the use of the kernel function unlike other behavioral models. This model strategy can also be applied to coupled engineering applications, such as the behavior of swarms of autonomous drones [27].

## Supporting information

S1 File Model code. Matlab code to run dynamic huddling model using coupled SPH-FVM.
(DOCX)
