# Supplementary material for: A novel coupled fluid-behavior model for simulating dynamic huddle formation
Source: PLoS One. 2018 Aug 31;13(8):e0203231. doi: 10.1371/journal.pone.0203231 (PMC6118378; doi:10.1371/journal.pone.0203231)
Supplement: S1 File — Matlab code to run dynamic huddling model using coupled SPH-FVM. (DOCX) [file pone.0203231.s001.docx]

**Supplementary Information**

**Model Code**

clear all

close all

clc

% Defining grid in the horizontal direction and vertical direction and

% calculate the x step and y step

xmin = 0.0; xmax = 50.0; xnum = 200.0;

xstp =(xmax-xmin)/xnum;

ymin = 0.0; ymax = 100.0; ynum = 400.0;

ystp =(ymax-ymin)/ynum;

Np = 223; % Number of particles

UX = zeros(xnum,ynum); % Current velocity matrix

UY = zeros(xnum,ynum);

Dist = zeros(xnum,ynum);

V = zeros(xnum,ynum); % total wind speed in each grid point

P = zeros(xnum,ynum); % Pressure variable matrix

p = zeros(xnum,ynum);

xp = zeros(1,Np); % Particle positions

yp = zeros(Np,1); % Particle positions

dx = 8; % Define length between particles

hp = 5; % Particle smoothness value

hhp =1;

tol = 1; % Check the mass conservation shold be less than this tolerance

DistToObj = zeros(1,Np);

load xp; % Load saved particle position and initial wind field

load yp;

% Defining Related Coefficient

D = 1.0; % Diffusion coefficient in m^2/s

timestep = 0.002; % Time step not to exceed 0.2 seconds for 1 m grid

Vel = 5 ; % Initial wind velocity in m/s

rhoair = 1.4224; % Density(kg/m3) of air at -25 degree C

mass = 50; % Penguin mass (constant)

height = 1.2; % Average height of adult emperor penguin

C = 0.1040; % Thermal conductance of penguin (W/C)

CA = 0.0204 ; % Thermal conductivity of air (W/mC)at-50 degree C

RadObj = 1; % Radius of the impediment object in grid size

k = .08; % Ideal gas equation Constance

a = 1; % File number

n = zeros(1,Np); % Initial number of particle within smooth length circle at the point of each particle

rhoparticle = zeros(1,Np); % Particle density

rhop = zeros(1,Np); % Density of the particle smooth circle

rhop0 = zeros(1,Np); % Initial particle density

rhopr = zeros(xnum,ynum); % Particle density at grid point

Ep = zeros(1,Np); % Local Metabolic energy within smooth length

Epr = zeros(xnum,ynum); % Local metabolic energy at grid point

Ei = zeros(1,Np); % Particle metabolic energy

Eir = zeros(xnum,ynum); % Particle metabolic energy at grid point

distance = zeros(1,Np);

DistToB1 = zeros(1,xnum); % Distance to boundaries

DistToB2 = zeros(1,xnum);

DistToB3 = zeros(1,ynum);

DistToB4 = zeros(1,ynum);

Tw = zeros(xnum,ynum); % Wind chill temperature

Tp = zeros(1,Np)+273.15; % Particle wind chill temperature in K

Pe = zeros(1,Np); % Particle pressure

fp = zeros(1,Np); % pressure acceleration

up = zeros(1,Np); % Particle velocity

r = zeros(Np,Np); % Distance between each particle

s = zeros(1,Np); % Particle moving distance

delT = zeros(1,Np); % Temperature change

delTr = zeros(xnum,ynum); % Temperature change at grid point

zeta = zeros(1,Np); % Moving angle

coszeta1 = zeros(Np,1);

sinzeta1 = zeros(Np,1);

coszeta2 = zeros(Np,1);

sinzeta2 = zeros(Np,1);

w = zeros(1,Np); % Kernel function

dw = zeros(1,Np);

ww = zeros(1,Np); % Kernel function

dww = zeros(1,Np);

Ti = ones(1,Np)*37.7; % Penguin body temperature is 37.7 degree C

Ta = -30*ones(xnum,ynum); % Ambient temperature

volqr = 0.345;

% Initialize partile on the grid

xexact=xp; % x position of penguins at t=0

yexact=yp; % y position of penguins at t=0

% Defining the maximum number of iterations for convergence

maxiter = 300000;

for iter = 1:maxiter

iter % iteration numbers

err = 0.0;

% Define the boundary condition for the current velocity matrices

% Set upwind x-boundary to normal velocity of applied wind

UX0 = UX; % Previous time velcoity matrix

UY0 = UY;

% Populate the pressure matrix using velocites from previous step

Lp = kron(speye(ynum),K1(xnum,xstp,1))+kron(K1(ynum,ystp,1),speye(xnum));

Lp(1,1) = 3/2*Lp(1,1);

perp = symamd(Lp); Rp = chol(Lp(perp,perp)); Rpt = Rp';

% upper boundary velocity update

UX(1,:) = 0;

UY(1,:) = Vel;

Tw(1,:) = 13.12 + 0.6215.*Ta(1,:) - 11.37*Vel^0.16 + 0.3965.*Ta(1,:)*Vel^0.16;

% Calculate wind velocity at interior points for x and y fr om 2 to (max-1)

if (iter ==1 ||iter == maxiter)

for xn = 2:xnum-1

for yn = 2:ynum-1

for ip = 1:Np

DistToObj(ip) = sqrt((xn-xp(ip))^2+(yn-yp(ip))^2) ; %Distance from current grid point to impeding object 1

Dist(xn,yn) = min(DistToObj);

end

end

end

%DistToObj1 = sqrt((xn-ObjX1)^2+(yn-ObjY1)^2);

for xn = 2:xnum-1

for yn = 2:ynum-1

Ta = -30*ones(xnum,ynum);

if Dist(xn,yn) <= RadObj % If the grid point is covered by an object, define the velocity and pressure to be zero

UX(xn,yn) = 0;

UY(xn,yn) = 0;

Tw(xn,yn) = Ta(xn,yn);

else

U2XY = (UX0(xn-1,yn)-2.0*UX0(xn,yn)+UX0(xn+1,yn))/(ystp)^2; %Central difference of 2nd derivative of x-velocity in x-direction

U2XX = (UX0(xn,yn-1)-2.0*UX0(xn,yn)+UX0(xn,yn+1))/(xstp)^2; %Central difference of 2nd derivative of x-velocity in y-direction

U1XY = (UX0(xn+1,yn)-UX0(xn-1,yn))/(2*ystp); %Central difference of 1st derivative of x-velcoity in x-direction

U1XX = (UX0(xn,yn+1)-UX0(xn,yn-1))/(2*xstp); %Central difference of 1st derivative of x-velcoity in y-direction

U2YX = (UY0(xn,yn-1)-2.0*UY0(xn,yn)+UY0(xn,yn+1))/(xstp)^2; %Central difference of 2nd derivative of y-velocity in y-direction

U2YY = (UY0(xn-1,yn)-2.0*UY0(xn,yn)+UY0(xn+1,yn))/(ystp)^2; %Central difference of 2nd derivative of y-velocity in x-direction

U1YX = (UY0(xn,yn+1)-UY0(xn,yn-1))/(2*xstp); %Central difference of 1st derivative of y-velcoity in y-direction

U1YY = (UY0(xn+1,yn)-UY0(xn-1,yn))/(2*ystp); %Central difference of 1st derivative of y-velocity in x-direction

UX(xn,yn) = UX0(xn,yn) + timestep*(D*(U2XX + U2XY) - UX0(xn,yn)*U1XX - UY0(xn,yn)*U1XY);

UY(xn,yn) = UY0(xn,yn) + timestep*(D*(U2YY + U2YX) - UY0(xn,yn)*U1YY - UX0(xn,yn)*U1YX);

U1XXcurrent = (UX(xn,yn)-UX(xn,yn-1))/xstp;

U1YYcurrent = (UY(xn,yn)-UY(xn-1,yn))/ystp;

err = max(err,timestep*abs(U1XXcurrent+U1YYcurrent));

V(xn,yn) = sqrt(UX(xn,yn)^2 + UY(xn,yn)^2);

end

end % End inner loop

end %outer loop

% left and right boudanry velocity update

for xn = 2: xnum-1

UX(xn,1) = 0;

UX(xn,ynum) = 0;

U2YXy1 = (UY0(xn,1)-2.0*UY0(xn,2)+UY0(xn,3))/(xstp)^2; %Forward difference of 2nd derivative of y-velocity in y-direction

U2YYy1 = (UY0(xn-1,1)-2.0*UY0(xn,1)+UY0(xn+1,1))/(ystp)^2; %Central difference of 2nd derivative of y-velocity in x-direction

U1YXy1 = (UY0(xn,2)-UY0(xn,1))/xstp; %Forward difference of 1st derivative of y-velcoity in y-direction

U1YYy1 = (UY0(xn+1,1)-UY0(xn-1,1))/(2*ystp); %Central difference of 1st derivative of y-velocity in x-direction

U2YXynum = (UY0(xn,ynum)-2.0*UY0(xn,ynum-1)+UY0(xn,ynum-2))/(xstp)^2; %Back difference of 2nd derivative of y-velocity in y-direction

U2YYynum = (UY0(xn-1,ynum)-2.0*UY0(xn,ynum)+UY0(xn+1,ynum))/(ystp)^2; %Central difference of 2nd derivative of y-velocity in x-direction

U1YXynum = (UY0(xn,ynum)-UY0(xn,ynum-1))/xstp; %Back difference of 1st derivative of y-velcoity in y-direction

U1YYynum = (UY0(xn+1,ynum)-UY0(xn-1,ynum))/(2*ystp); %Central difference of 1st derivative of y-velocity in x-direction

UY(xn,1) = UY0(xn,1) + timestep*(D*(U2YYy1 + U2YXy1) - UY0(xn,1)*U1YYy1 - UX0(xn,1)*U1YXy1);

UY(xn,ynum) =UY0(xn,ynum) + timestep*(D*(U2YYynum + U2YXynum) - UY0(xn,ynum)*U1YYynum - UX0(xn,ynum)*U1YXynum);

V(xn,1) = sqrt(UX(xn,1)^2 + UY(xn,1)^2);

% Calculate average wind speed affecting particle

if V(xn,1) >= 1.3

Tw(xn,1) = 13.12 + 0.6215.*Ta(xn,1) - 11.37.*V(xn,1).^0.16 + 0.3965.*Ta(xn,1).*V(xn,1).^0.16;

else

Tw(xn,1) = Ta(xn,1);

end

V(xn,ynum) = sqrt(UX(xn,ynum)^2 + UY(xn,ynum)^2);

% Calculate average wind speed affecting particle

if V(xn,ynum) >= 1.3

Tw(xn,ynum) = 13.12 + 0.6215.*Ta(xn,ynum) - 11.37.*V(xn,ynum).^0.16 + 0.3965.*Ta(xn,ynum).*V(xn,ynum).^0.16;

else

Tw(xn,ynum) = Ta(xn,ynum);

end

end

% bottom boundary velocity update

for yn = 2: ynum-1

U2XXxum = (UX0(xnum,yn+1)-2.0*UX0(xnum,yn)+UX0(xnum,yn-1))/(xstp)^2; %Central difference of 2nd derivative of y-velocity in y-direction

U2XYxum = (UX0(xnum-2,yn)-2.0*UX0(xnum-1,yn)+UX0(xnum,yn))/(ystp)^2; %Central difference of 2nd derivative of y-velocity in x-direction

U1XXxum = (UX0(xnum,yn+1)-UX0(xnum,yn-1))/(2*xstp); %Central difference of 1st derivative of y-velcoity in y-direction

U1XYxum = (UX0(xnum,yn)-UX0(xnum-1,yn))/(ystp); %Central difference of 1st derivative of y-velocity in x-direction

U2YYxum = (UY0(xnum-2,yn)-2.0*UY0(xnum-1,yn)+UY0(xnum,yn))/(ystp)^2; %Central difference of 2nd derivative of x-velocity in x-direction

U2YXxum = (UY0(xnum,yn+1)-2.0*UY0(xnum,yn)+UY0(xnum,yn-1))/(xstp)^2; %Central difference of 2nd derivative of x-velocity in y-direction

U1YYxum = (UY0(xnum,yn)-UY0(xnum-1,yn))/(ystp); %Central difference of 1st derivative of x-velcoity in x-direction

U1YXxum = (UX0(xnum,yn+1)-UX0(xnum,yn-1))/(2*xstp); %Central difference of 1st derivative of x-velcoity in y-direction

UY(xnum,yn) = UY0(xnum,yn) + timestep*(D*(U2YYxum + U2YXxum) - UY0(xnum,yn)*U1YYxum - UX0(xnum,yn)*U1YXxum );

UX(xnum,yn) = UX0(xnum,yn) + timestep*(D*(U2XXxum + U2XYxum) - UX0(xnum,yn)*U1XXxum - UY0(xnum,yn)*U1XYxum );

V(xnum,yn) = sqrt(UX(xnum,yn)^2 + UY(xnum,yn)^2);

% Calculate average wind speed affecting particle

if V(xnum,yn) >= 1.3

Tw(xnum,yn) = 13.12 + 0.6215.*Ta(xnum,yn) - 11.37.*V(xnum,yn).^0.16 + 0.3965.*Ta(xnum,yn).*V(xnum,yn).^0.16;

else

Tw(xnum,yn) = Ta(xnum,yn);

end

end

% Left Bottom Boundary point and right Bottem boundary point

UX(xnum,ynum)=0;

UX(xnum,1) = 0;

U1XX1 = (UX0(xnum,2)-UX0(xnum,1))/xstp;

U1YY1 = U1XX1;

UY(xnum,1) = U1YY1*ystp+ UY(xnum-1,1);

U1XXnum =(UX0(xnum,ynum)-UX0(xnum,ynum-1))/xstp;

U1YYnum = U1XXnum;

UY(xnum,ynum) = U1YYnum*ystp+ UY(xnum-1,ynum);

V(xnum,ynum) = sqrt(UX(xnum,ynum)^2 + UY(xnum,ynum)^2); % Calculate average wind speed affecting particle

if V(xnum,ynum) >= 1.3

Tw(xnum,ynum) = 13.12 + 0.6215.*Ta(xnum,ynum) - 11.37.*V(xnum,ynum).^0.16 + 0.3965.*Ta(xnum,ynum).*V(xnum,ynum).^0.16;

else

Tw(xnum,ynum) = Ta(xnum,ynum);

end

V(xnum,1) = sqrt(UX(xnum,1)^2 + UY(xnum,1)^2);

% Calculate average wind speed affecting particle

if V(xnum,1) >= 1.3

Tw(xnum,1) = 13.12 + 0.6215.*Ta(xnum,1) - 11.37.*V(xnum,1).^0.16 + 0.3965.*Ta(xnum,1).*V(xnum,1).^0.16;

else

Tw(xnum,1) = Ta(xnum,1);

end

%Pressure correction

AA = UY(:,1);

AB = UX(xnum,:);

rhs = reshape((diff([UX;AB])/xstp+diff([AA';UY'])'/ystp)*timestep,[],1);

p(perp) = (-Rp\(Rpt\rhs(perp)));

P = reshape(p,xnum,ynum);

P(1,1) = 3/2*P(1,1);

if iter >5000

for i = 1:35

for j = 1:25

if P(i,j)>P(36,1)

P(i,j)=P(36,1);

end

end

end

end

AC = diff(P);

AD = diff(P')';

AC(:,1)=0;

AC(:,end)=0;

AD(1,:) = 0;

UX(2:end,:) = UX(2:end,: )-AC/xstp*0.8;

UY(:,1:end-1) = UY(:,1:end-1)-AD*0.8;

end

if (iter ==1 ||mod(iter,15000)==0)

Epr = zeros(xnum,ynum);

Eir = zeros(1,Np);

rhopr = zeros(xnum,ynum);

rr = zeros(1,Np);

drr = zeros(1,Np);

m = zeros(xn,yn);

n = zeros(1,Np);

Pe = zeros(1,Np); % Particle pressure

bpX = zeros(1,Np);

bpY = zeros(1,Np);

upX = zeros(1,Np); % Particle velocity in x direction

upY = zeros(1,Np); % Particle velocity in y direction

bp = zeros(1,Np);

fp = zeros(1,Np);

fpX = zeros(1,Np); % pressure acceleration in x direction

fpY = zeros(1,Np); % pressure acceleration in y direction

rhoparticle = zeros(1,Np);

Ep = zeros(1,Np);

Tp = zeros (1,Np);

volq = zeros(1,Np);

Ei = zeros(1,Np);

A = zeros(1,Np);

Ta = -30*ones(xnum,ynum); % Ambient temperature

for xn = 1:xnum

for yn = 1:ynum

for ip=1:Np

for iq=1:Np

if iq==ip

continue;

end

xpq = xp(ip)-xp(iq) ;

ypq = yp(ip)-yp(iq);

r(ip,iq) = sqrt(xpq^2+ypq^2);

hhpq = hhp;

[w(ip,iq),dw(ip,iq),stopIter] = penguin_kernel(hhpq,r(ip,iq));

if w(ip,iq)>0

n(ip) = n(ip)+1;

end

end

end

for ip = 1:Np

xa = xn-xp(ip) ;

ya = yn-yp(ip);

ha = sqrt(xa^2+ya^2);

hpq = hp;

[rr(ip),drr(ip),stopIter] = penguin_kernel(hpq,ha);

if (rr(ip)>0)

Eir(ip) = C*(Ti(ip)-Ta(xn,yn));

Epr(xn,yn) = Epr(xn,yn) + (Eir(ip)*volqr*rr(ip));

else

Epr(xn,yn) = Epr(xn,yn);

end

end

delTr(xn,yn) = Epr(xn,yn)./(CA*rhoair*pi*hp^2*height);

Ta(xn,yn) = Ta(xn,yn) + delTr(xn,yn);

end

end

for xn = 2:xnum-1

for yn = 2:ynum-1

T1X = (Ta(xn,yn+1)-Ta(xn,yn-1))/(2*xstp);

T1Y = (Ta(xn+1,yn)-Ta(xn-1,yn))/(2*ystp);

T2Y = (Ta(xn-1,yn)-2.0*Ta(xn,yn)+Ta(xn+1,yn))/(ystp)^2;

T2X = (Ta(xn,yn-1)-2.0*Ta(xn,yn)+Ta(xn,yn+1))/(xstp)^2;

Ta(xn,yn)= Ta(xn,yn) + timestep*(D*(T2X + T2Y) - UX(xn,yn)*T1X - UY(xn,yn)*T1Y);

if V(xn,yn)>1.3

Tw(xn,yn) = 13.12 + 0.6215.*Ta(xn,yn) - 11.37.*(3.6*V(xn,yn)).^0.16 + 0.3965.*Ta(xn,yn).*(3.6*V(xn,yn)).^0.16;

else

Tw(xn,yn) = Ta(xn,yn);

end

end

end

for ip=1:Np

Vtem =[V(floor(xp(ip))-2,floor(yp(ip))-2),V(floor(xp(ip))-2,ceil(yp(ip))+2),V(ceil(xp(ip))+2,floor(yp(ip))-2),V(ceil(xp(ip))+2,ceil(yp(ip))+2)];

if max(Vtem)>1.3

Twtem = [Tw(floor(xp(ip))-2,floor(yp(ip))-2),Tw(floor(xp(ip))-2,ceil(yp(ip))+2),Tw(ceil(xp(ip))+2,floor(yp(ip))-2),Tw(ceil(xp(ip))+2,ceil(yp(ip))+2)];

Tp(ip)= mean(Twtem)+273.15;

else

Tp(ip) = (Tw(floor(xp(ip)),floor(yp(ip)))+Tw(floor(xp(ip)),ceil(yp(ip)))+Tw(ceil(xp(ip)),floor(yp(ip)))+Tw(ceil(xp(ip)),ceil(yp(ip))))/4+273.15;

end

end

for ip = 1:Np

CenterX = sum(xp)/Np;

CenterY = sum(yp)/Np;

if xp(ip)-CenterX ~=0

coszeta1(ip)= (CenterX-xp(ip))/(sqrt(((xp(ip)-CenterX)^2)+((yp(ip)-CenterY)^2)));

sinzeta1(ip)= (CenterY-yp(ip))/(sqrt(((xp(ip)-CenterX)^2)+((yp(ip)-CenterY)^2)));

end

if Tp(ip) <263.15

bp(ip) = (-0.2354*Tp(ip)+62.507)*0.9;

bpX(ip) = bp(ip)*coszeta1(ip);

bpY(ip) = bp(ip)*sinzeta1(ip);

else

bpX(ip) = 0;

bpY(ip) =0;

end

for iq = 1: Np

if (iq==ip)

continue;

else

if w(ip,iq)>0

Pe = ones(1,Np).*0.1;

density = mass/(pi*RadObj^2*height);

fpX(ip) = fpX(ip) - mass.*((Pe(ip)/density.^2)+(Pe(iq)./density.^2)).*(xp(ip)-xp(iq))/abs(r(ip,iq)).*dw(ip,iq)*10;

fpY(ip) = fpY(ip) - mass.*((Pe(ip)/density.^2)+(Pe(iq)./density.^2)).*(yp(ip)-yp(iq))/abs(r(ip,iq)).*dw(ip,iq)*10;

end

end

end

upX(ip) = 50*timestep*(bpX(ip)+fpX(ip));

upY(ip) = 50*timestep*(bpY(ip)+fpY(ip));

end

end

for ip = 1:Np

if xp(ip) > xnum-1

xp(ip) = xp(ip) - xnum +2;

elseif yp(ip)> ynum-1

yp(ip) = yp(ip) - ynum +2;

elseif xp(ip) < 1

xp(ip) = xp(ip) + xnum -1;

elseif yp(ip) < 1

yp(ip) = yp(ip) + ynum-1 ;

end

xp(ip) = xp(ip) + upX(ip).*timestep; % Relocate new position of particle

yp(ip) = yp(ip) + upY(ip).*timestep;

UX(round(xp(ip)),round(yp(ip)))=0;

UY(round(xp(ip)),round(yp(ip)))=0;

end

err

UX0=UX;% Resetting current values for the next iteration

UY0=UY;

if (iter ==1 ||iter == maxiter)

aberrorX = abs(UX0-UX);

aberrorY = abs(UY0-UY);

maxaberrorX = max(max(aberrorX));

maxaberrorY = max(max(aberrorY));

while max(maxaberrorX,maxaberrorY)> 0.05

for xn = 2:xnum-1

for yn = 2:ynum-1

Ta = -30*ones(xnum,ynum);

if Dist(xn,yn) <= RadObj % If the grid point is covered by an object, define the velocity and pressure to be zero

UX(xn,yn) = 0;

UY(xn,yn) = 0;

Tw(xn,yn) = Ta(xn,yn);

else

U2XY = (UX0(xn-1,yn)-2.0*UX0(xn,yn)+UX0(xn+1,yn))/(ystp)^2; %Central difference of 2nd derivative of x-velocity in x-direction

U2XX = (UX0(xn,yn-1)-2.0*UX0(xn,yn)+UX0(xn,yn+1))/(xstp)^2; %Central difference of 2nd derivative of x-velocity in y-direction

U1XY = (UX0(xn+1,yn)-UX0(xn-1,yn))/(2*ystp); %Central difference of 1st derivative of x-velcoity in x-direction

U1XX = (UX0(xn,yn+1)-UX0(xn,yn-1))/(2*xstp); %Central difference of 1st derivative of x-velcoity in y-direction

U2YX = (UY0(xn,yn-1)-2.0*UY0(xn,yn)+UY0(xn,yn+1))/(xstp)^2; %Central difference of 2nd derivative of y-velocity in y-direction

U2YY = (UY0(xn-1,yn)-2.0*UY0(xn,yn)+UY0(xn+1,yn))/(ystp)^2; %Central difference of 2nd derivative of y-velocity in x-direction

U1YX = (UY0(xn,yn+1)-UY0(xn,yn-1))/(2*xstp); %Central difference of 1st derivative of y-velcoity in y-direction

U1YY = (UY0(xn+1,yn)-UY0(xn-1,yn))/(2*ystp); %Central difference of 1st derivative of y-velocity in x-direction

UX(xn,yn) = UX0(xn,yn) + timestep*(D*(U2XX + U2XY) - UX0(xn,yn)*U1XX - UY0(xn,yn)*U1XY);

UY(xn,yn) = UY0(xn,yn) + timestep*(D*(U2YY + U2YX) - UY0(xn,yn)*U1YY - UX0(xn,yn)*U1YX);

U1XXcurrent = (UX(xn,yn)-UX(xn,yn-1))/xstp;

U1YYcurrent = (UY(xn,yn)-UY(xn-1,yn))/ystp;

err = max(err,timestep*abs(U1XXcurrent+U1YYcurrent));

V(xn,yn) = sqrt(UX(xn,yn)^2 + UY(xn,yn)^2); % Calculate average wind speed affecting particle

if V(xn,yn) >= 1.3

Tw(xn,yn) = 13.12 + 0.6215.*Ta(xn,yn) - 11.37.*V(xn,yn).^0.16 + 0.3965.*Ta(xn,yn).*V(xn,yn).^0.16;

else

Tw(xn,yn) = Ta(xn,yn);

end

end

end % End inner loop

end %outer loop

% left and right boudanry velocity update

for xn = 2: xnum-1

UX(xn,1) = 0;

UX(xn,ynum) = 0;

U2YXy1 = (UY0(xn,1)-2.0*UY0(xn,2)+UY0(xn,3))/(xstp)^2; %Forward difference of 2nd derivative of y-velocity in y-direction

U2YYy1 = (UY0(xn-1,1)-2.0*UY0(xn,1)+UY0(xn+1,1))/(ystp)^2; %Central difference of 2nd derivative of y-velocity in x-direction

U1YXy1 = (UY0(xn,2)-UY0(xn,1))/xstp; %Forward difference of 1st derivative of y-velcoity in y-direction

U1YYy1 = (UY0(xn+1,1)-UY0(xn-1,1))/(2*ystp); %Central difference of 1st derivative of y-velocity in x-direction

U2YXynum = (UY0(xn,ynum)-2.0*UY0(xn,ynum-1)+UY0(xn,ynum-2))/(xstp)^2; %Back difference of 2nd derivative of y-velocity in y-direction

U2YYynum = (UY0(xn-1,ynum)-2.0*UY0(xn,ynum)+UY0(xn+1,ynum))/(ystp)^2; %Central difference of 2nd derivative of y-velocity in x-direction

U1YXynum = (UY0(xn,ynum)-UY0(xn,ynum-1))/xstp; %Back difference of 1st derivative of y-velcoity in y-direction

U1YYynum = (UY0(xn+1,ynum)-UY0(xn-1,ynum))/(2*ystp); %Central difference of 1st derivative of y-velocity in x-direction

UY(xn,1) = UY0(xn,1) + timestep*(D*(U2YYy1 + U2YXy1) - UY0(xn,1)*U1YYy1 - UX0(xn,1)*U1YXy1);

UY(xn,ynum) =UY0(xn,ynum) + timestep*(D*(U2YYynum + U2YXynum) - UY0(xn,ynum)*U1YYynum - UX0(xn,ynum)*U1YXynum);

V(xn,1) = sqrt(UX(xn,1)^2 + UY(xn,1)^2); % Calculate average wind speed affecting particle

if V(xn,1) >= 1.3

Tw(xn,1) = 13.12 + 0.6215.*Ta(xn,1) - 11.37.*V(xn,1).^0.16 + 0.3965.*Ta(xn,1).*V(xn,1).^0.16;

else

Tw(xn,1) = Ta(xn,1);

end

V(xn,ynum) = sqrt(UX(xn,ynum)^2 + UY(xn,ynum)^2); % Calculate average wind speed affecting particle

if V(xn,ynum) >= 1.3

Tw(xn,ynum) = 13.12 + 0.6215.*Ta(xn,ynum) - 11.37.*V(xn,ynum).^0.16 + 0.3965.*Ta(xn,ynum).*V(xn,ynum).^0.16;

else

Tw(xn,ynum) = Ta(xn,ynum);

end

end

% bottom boundary velocity update

for yn = 2: ynum-1

U2XXxum = (UX0(xnum,yn+1)-2.0*UX0(xnum,yn)+UX0(xnum,yn-1))/(xstp)^2; %Central difference of 2nd derivative of y-velocity in y-direction

U2XYxum = (UX0(xnum-2,yn)-2.0*UX0(xnum-1,yn)+UX0(xnum,yn))/(ystp)^2; %Central difference of 2nd derivative of y-velocity in x-direction

U1XXxum = (UX0(xnum,yn+1)-UX0(xnum,yn-1))/(2*xstp); %Central difference of 1st derivative of y-velcoity in y-direction

U1XYxum = (UX0(xnum,yn)-UX0(xnum-1,yn))/(ystp); %Central difference of 1st derivative of y-velocity in x-direction

U2YYxum = (UY0(xnum-2,yn)-2.0*UY0(xnum-1,yn)+UY0(xnum,yn))/(ystp)^2; %Central difference of 2nd derivative of x-velocity in x-direction

U2YXxum = (UY0(xnum,yn+1)-2.0*UY0(xnum,yn)+UY0(xnum,yn-1))/(xstp)^2; %Central difference of 2nd derivative of x-velocity in y-direction

U1YYxum = (UY0(xnum,yn)-UY0(xnum-1,yn))/(ystp); %Central difference of 1st derivative of x-velcoity in x-direction

U1YXxum = (UX0(xnum,yn+1)-UX0(xnum,yn-1))/(2*xstp); %Central difference of 1st derivative of x-velcoity in y-direction

UY(xnum,yn) = UY0(xnum,yn) + timestep*(D*(U2YYxum + U2YXxum) - UY0(xnum,yn)*U1YYxum - UX0(xnum,yn)*U1YXxum );

UX(xnum,yn) = UX0(xnum,yn) + timestep*(D*(U2XXxum + U2XYxum) - UX0(xnum,yn)*U1XXxum - UY0(xnum,yn)*U1XYxum );

V(xnum,yn) = sqrt(UX(xnum,yn)^2 + UY(xnum,yn)^2); % Calculate average wind speed affecting particle

if V(xnum,yn) >= 1.3

Tw(xnum,yn) = 13.12 + 0.6215.*Ta(xnum,yn) - 11.37.*V(xnum,yn).^0.16 + 0.3965.*Ta(xnum,yn).*V(xnum,yn).^0.16;

else

Tw(xnum,yn) = Ta(xnum,yn);

end

end

% Left Bottom Boundary point and right Bottem boundary point

UX(xnum,ynum)=0;

UX(xnum,1) = 0;

U1XX1 = (UX0(xnum,2)-UX0(xnum,1))/xstp;

U1YY1 = U1XX1;

UY(xnum,1) = U1YY1*ystp+ UY(xnum-1,1);

U1XXnum =(UX0(xnum,ynum)-UX0(xnum,ynum-1))/xstp;

U1YYnum = U1XXnum;

UY(xnum,ynum) = U1YYnum*ystp+ UY(xnum-1,ynum);

V(xnum,ynum) = sqrt(UX(xnum,ynum)^2 + UY(xnum,ynum)^2); % Calculate average wind speed affecting particle

if V(xnum,ynum) >= 1.3

Tw(xnum,ynum) = 13.12 + 0.6215.*Ta(xnum,ynum) - 11.37.*V(xnum,ynum).^0.16 + 0.3965.*Ta(xnum,ynum).*V(xnum,ynum).^0.16;

else

Tw(xnum,ynum) = Ta(xnum,ynum);

end

V(xnum,1) = sqrt(UX(xnum,1)^2 + UY(xnum,1)^2); % Calculate average wind speed affecting particle

if V(xnum,1) >= 1.3

Tw(xnum,1) = 13.12 + 0.6215.*Ta(xnum,1) - 11.37.*V(xnum,1).^0.16 + 0.3965.*Ta(xnum,1).*V(xnum,1).^0.16;

else

Tw(xnum,1) = Ta(xnum,1);

end

%Pressure correction

AA = UY(:,1);

AB = UX(xnum,:);

rhs = reshape((diff([UX;AB])/xstp+diff([AA';UY'])'/ystp)*timestep,[],1);

p(perp) = (-Rp\(Rpt\rhs(perp)));

P = reshape(p,xnum,ynum);

P(1,1) = 3/2*P(1,1);

if iter >5000

for i = 1:35

for j = 1:25

if P(i,j)>P(36,1)

P(i,j)=P(36,1);

end

end

end

end

AC = diff(P);

AD = diff(P')';

AC(:,1)=0;

AC(:,end)=0;

AD(1,:) = 0;

UX(2:end,:) = UX(2:end,: )-AC/xstp;

UY(:,1:end-1) = UY(:,1:end-1)-AD;

end

end

if (mod(iter,300000)==0)

filename = [ '20180316' num2str(a) '.mat' ];

save(filename);

a = a+1;

end

end

plot(xp,yp,'or','MarkerSize',5); grid on; xlim([0 200]);ylim([0 400]); drawnow;

subplot(1,2,1),surf(UX), xlabel('X'),ylabel('Y');

subplot(1,2,2),surf(UY), xlabel('X'),ylabel('Y');

x = xmin:xstp:xmax;

y = ymin:ystp:ymax;

set(gcf,'Renderer','zbuffer');
